# A Modified Complex-Valued Encoding Greater Cane Rat Algorithm for Global Optimization and Constrained Engineering Applications

**DOI:** 10.3390/biomimetics11060413

**Published:** 2026-06-11

**Authors:** Yubao Xu, Yuebo Wu, Jinzhong Zhang

**Affiliations:** School of Electrical and Photoelectronic Engineering, West Anhui University, Lu’an 237012, China; xuyubao@wxc.edu.cn (Y.X.); 42000021@wxc.edu.cn (Y.W.)

**Keywords:** greater cane rat algorithm, complex-valued encoding, benchmark functions, engineering designs, exploration and exploitation

## Abstract

The greater cane rat algorithm (GCRA) draws inspiration from the seasonal behavioral patterns of the greater cane rats: extensive roaming during the non-breeding period for global exploration, and aggregative foraging during the reproductive period for local exploitation. The GCRA leverages independent movement and population aggregation to iteratively update positions in pursuit of the optimal solution, which exhibits inherent structural deficiencies: precipitous population diversity collapse, lethargic convergence dynamics, suboptimal computational precision, high susceptibility to local optima, and severe dimensional scalability. This paper proposes a modified complex-valued encoding GCRA (CGCRA) that exploits the mathematical structure of complex numbers to construct a two-dimensional search domain on the complex plane and facilitate collaborative optimization. The CGCRA maps the decision variables onto the complex domain, the real part executes the native foraging mechanism for local fine-grained exploitation, and the imaginary part exploits phase rotation to generate global exploratory perturbations. The CGCRA leverages a dual-encoding redundancy mechanism with inherent error tolerance to attenuate result volatility, augment information capacity and population heterogeneity, elevate search adaptability and disturbance rejection, accelerate parallel computation and exploration efficiency, and facilitate spatial transformation and multi-dimensional data manipulation. Twenty-three benchmark functions and twelve real-world engineering designs are employed to assess the CGCRA’s stability and practical feasibility rigorously. The CGCRA delivers comprehensive spatial mapping and adaptive coordination to facilitate population collaboration and bolster resilience, expedite exhaustive research, and advance optimization efficiency. The experimental results demonstrate that the CGCRA emphasizes instructive superiority and practical utility to regulate exploration and exploitation, reduce result dispersion, mitigate search stagnation, accelerate convergence efficiency, elevate solution precision, and fortify stability and robustness.

## 1. Introduction

The optimization problem comprises an objective function, decision variables, and constraints. The purpose is to accomplish the objective value by systematically altering the decision variables in the solution zone through specific search strategies and iterative mechanisms. Numerous complex issues with multi-model construction, multi-objective optimization, multi-dimensionality, multi-factors, large-scale integration, high comprehensiveness, uncertainty and dynamics, and high computational complexity must be tackled urgently. The traditional methods exhibit some limitations: rely on the issue’s specific structure and properties, lack a theoretical guarantee and prior knowledge for global optimization, require different parameter tuning and dynamic change, easy to trap in local optimality, sensitive to initial solution, poor scalability and repeatability, easy combination explosion, slow detection efficiency, and low mining accuracy. However, metaheuristic algorithms (MAs) are an advanced algorithm framework designed based on the simulation and abstraction of natural phenomena, physical, chemistry, or mathematical properties, intelligent biological group behavior, or human cognitive patterns. MAs utilize some empirical, intuitive, and universal heuristic principles to iteratively search in the solution space, promote search efficiency, explore solution space, and determine a high-quality optimum solution, which exhibits certain advantages: good self-organization and anti-interference, strong stability and robustness, natural distribution and consistency, excellent fault tolerance and collaboration, strong adaptability and parallelism, harmonized global recognition and local refinement, simple implementation and extensibility, and robust intelligence and versatility, such as the black-winged kite algorithm (BKA) [1], eel and grouper optimization (EGO) [2], horned lizard optimization algorithm (HLOA) [3], information acquisition optimization (IAO) [4], Newton-Raphson-based optimization (NRBO) [5], secretary bird optimization algorithm (SBOA) [6], wave search algorithm (WSA) [7], elk herd optimization (EHO) [8], walrus optimization (WO) [9], human evolutionary optimization algorithm (HEOA) [10], puma optimization (PO) [11], and greater cane rat algorithm (GCRA) [12]. The MAs are classified into four categories based on different structural principles and detection search strategies: swarm intelligence, evolutionary computation, physics/chemistry/mathematics-inspired optimizers, and human behavior-inspired approaches. Table 1 delineates the cross-dimensional analysis of four categories.

Bálint et al. employed molecular descriptors as quantitative evaluation proxies to conduct a systematic comparative investigation of multiple molecular geometry optimization methodologies. The proposed algorithm-established evaluation metrics by extracting geometric, energy, and topological descriptors, subsequently performed a quantitative comparative analysis of the convergence precision, computational efficiency, and configuration fidelity across various algorithms, and delineated the molecular systems and application boundaries [13]. Sörensen et al. rigorously deconstructed the phenomenon of metaphor abuse in metaheuristic algorithm design; contemporary research exhibited a troubling tendency to prioritize biological concepts and superficial packaging and neglect mathematical rigor and substantive innovation. The article emphasized that metaphors can inspire but cannot replace tangible improvements in search operators and algorithmic logic to cause research homogenization. The merit, innovation, and comprehensive efficacy of algorithms must be rigorously substantiated through mathematical mechanism analysis, convergence proofs, standardized performance testing, and practical engineering examples [14]. Miao et al. formulated a hybrid-strategy sand cat swarm optimization, signal decomposition, and neural fuzzy network to construct a dual-phase predictive model for estimating the health status of lithium-ion batteries. The proposed algorithm elevated the signal decomposition quality and model-fitting capacity, denoised raw monitoring data, extracted multi-scale degradation features, boosted prediction accuracy and resilience, and captured the nonlinear and non-stationary characteristics of battery aging signals [15]. Liu et al. constructed a hybrid forecasting framework that synergistically integrated entropy-guided decomposition with tuned neuro-fuzzy networks to forecast the urban air quality index accurately. The proposed algorithm employed information entropy to optimize network parameters, attenuate noise interference, and promote predictive precision [16]. Miao et al. merged enhanced fuzzy neural networks with fluid dynamics models to facilitate real-time trajectory navigation in dynamic spaces. The proposed algorithm leveraged the flow field characteristics and fuzzy neural networks to generate smooth trajectories, materialize adaptive parameter tuning, achieve rapid evasion of dynamic obstacles, and exhibit real-time responsiveness and operational safety [17]. Zhang et al. orchestrated a synergistic fusion among the GCRA, the sine–cosine algorithm’s inherent cyclical fluctuations, and the adaptive regulatory framework of a nonlinear control strategy to produce a multifaceted performance enhancement of superior detection efficiency, heightened mining precision, augmented search adaptability, and increased population heterogeneity. The proposed algorithm exhibited robust practicality and unwavering dependability to attain superior solution accuracy and convergence speed [18]. Chen et al. devised the GCRA underpinned by adaptive and global guidance mechanisms, specifically tailored for function optimization and engineering design. The proposed algorithm synergistically integrates a flexible parameter-tuning framework with a local disturbance operator to amplify the global optimal guidance component, sharpen convergence directionality, preclude search stagnation, yield high-quality solutions, expedite convergence efficiency, and elevate robustness and competitiveness [19]. Alshammari et al. integrated an artificially intelligent GCRA with the cross-language robust feature, which utilized the speech emotion identification technique to generate the associated spectrogram, designed the MobileNetv3 model to select the optimum hyperparameters, and adopted the extreme learning machine to identify the speech emotions. The proposed algorithm had strong multimodal fusion and dynamic robustness to avoid uneven distribution of language resources, eliminate the impact of language differences on emotional representation, and accurately recognize emotional states [20]. Malathi et al. introduced an integrated architecture for grid-connected photovoltaic converters. The proposed algorithm demonstrated remarkable adaptability for embedding control models and excellent performance, which achieved high energy conversion efficiency, maintained ultra-low total harmonic distortion, and boosted control accuracy and disturbance rejection capability [21]. Hu et al. constructed a short-term wind power forecasting framework with the maximal information coefficient, selecting the best and long-term memory that integrated the GCRA with the combined feature selection to screen key meteorological features and optimize model operating parameters dynamically. The proposed algorithm utilized the organic combination of dynamic feature dimensionality reduction and the metaheuristic algorithm, the collaborative optimization of multi-source feature screening and multi-modal data integration, and the mutual promotion of the multi-dimensional data fusion and the adaptive optimization mechanism to boost the forecasting precision and computational productivity, diminish unpredictability and noise interference broader power, and ensure dependability and supremacy [22]. AboRas et al. incorporated the GCRA into the foremost power point tracking scheme to address inherent deficiencies of oscillatory instability, tracking inaccuracies, insufficient steady-state precision, and weak disturbance resilience. The proposed algorithm exhibited formidable global discovery and refined local extraction to accurately locate the overall operating point, suppress steady-state power oscillations, and elevate the energy capture efficiency [23]. Karra et al. integrated the GCRA with a multi-source fusion extreme learning machine to formulate a fault-tolerant and flexible federated learning framework for unsupervised cross-domain face identification. The proposed algorithm was deployed to jointly tune network hyperparameters and fusion coefficients to neutralize random instability and local optimum susceptibility, which elevated feature fusion capability, cross-domain generalization, and system robustness under heterogeneous data distributions [24]. Ekinci et al. integrated the GCRA with a proportional-integral-double-derivative controller to counteract the pronounced large lag, severe nonlinearity, time-varying disturbances, and parameter tuning intractability for industrial electric furnace temperature control. The proposed algorithm rectified the deficiencies of empirical tuning, delayed the reaction, excessive overshoot, and oscillatory dynamics, and substantially elevated dynamic responsiveness and the steady-state regulation precision [25]. Isham et al. leveraged the GCRA to refine an extreme learning machine for intelligent fault diagnosis. The proposed algorithm exploited excellent global detection and local mining to rectify the deficiencies of suboptimal diagnostic accuracy, insufficient stability, and weak generalization, which exhibited marked competitiveness and inherent superiority to boost fault recognition accuracy and system stability in complex mechanical equipment operating environments [26]. To summarize, research endeavors concerning the GCRA are predominantly bifurcated into the algorithmic enhancement and practical deployment. (1) For algorithmic enhancement, the existing research on the GCRA predominantly revolved around four improvement avenues: adaptive parameter tuning, hybrid strategy integration, boundary-augmented population initialization, and discrete structural reconfiguration. These enhancements collectively strengthened comprehensive detection and mining, expedited convergence efficiency, attenuated iterative oscillations, elevated stability and robustness, and enriched population heterogeneity and search stagnation. Although the modifications have rectified certain deficiencies of the original GCRA, the enhanced version still exhibited substantial intrinsic limitations, such as solidification underlying the encoding scheme, the performance improvement ceiling, over-reliance on superficial modifications, insufficient fundamental innovation, poor strategy transferability, pronounced problem-specific dependence, expensive diversity preservation, and escalated algorithmic complexity. (2) For practical deployment, the GCRA has been successfully deployed across multiple domains that contained industrial control, energy optimization, fault diagnosis, intelligent recognition, route planning, and constrained engineering design, which was distinguished by structural simplicity, a minimal parameter set, and robust stability. Nevertheless, the GCRA exhibited discernible deficiencies when deployed in high-precision, highly coupled, and strongly constrained complex scenarios, such as the inadequate handling of high-dimensional complex functions, weak adaptability to multi-variable coupling engineering contexts, and restricted cross-domain applicability.

Although the altered versions of GCRA have demonstrated the flexibility and adaptability to accelerate detection efficiency and promote refined mining accuracy, there are still deficiencies in coordinating global investigation and local utilization. The no-free-lunch (NFL) stipulates that there is no superior approach with strong versatility and applicability to reconcile all sorts of difficulties. Each algorithm is characterized by the applicable scenarios and inherent limitations, which is necessary to select a reasonable algorithm based on its characteristics and structure to achieve the optimum high-quality solution. The GCRA is derived from the ingenious foraging patterns of the GCRs, which exhibit strong territorial awareness and high nocturnal activity to forage and leave the trails for food, water resources, and shelter. The CGCRA can overcome the drawbacks of languid convergence speed, inadequate calculation precision, susceptibility to local optimum, noteworthy dimensionality disaster, and a tendency towards imbalance between prospecting and mining. The CGCRA is explored to assess the global optimization; the aim is to quantitatively quantify the convergence productivity, resolution precision, stability and repeatability of the CGCRA by minimizing the fitness solution to comprehensively enhance the engineering quality and system reliability by optimizing the control variables and optimum solutions. The CGCRA exhibits not only strong information interaction and collaborative detection to enhance search flexibility and anti-interference ability, promote large-scale detection, and refined mining, and avoid search stagnation and local optimality, but also exhibits strong stability and repeatability to facilitate spatial transformation and multi-dimensional data processing, increase population diversity and exploration efficiency, and achieve the high-quality optimum solution and convergence accuracy.

The contributions of the CGCRA are delineated as follows: (1) the CGCRA is advocated to tackle global optimization. (2) The complex-valued encoding increases information capacity and population diversity, enhances search flexibility and anti-interference ability, promotes parallel computing and exploration efficiency, facilitates spatial transformation and multi-dimensional data processing, alleviates local optimality and random factors interference, and promotes consistency and repeatability. (3) The CGCRA is comprehensively evaluated against a multitude of comparison approaches, including newly published, extensively referenced, highly executed approaches, such as BKA, EGO, HLOA, IAO, NRBO, SBOA, WSA, EHO, WO, HEOA, PO, and GCRA. (4) The CGCRA is explored to resolve benchmark functions and engineering designs by minimizing the fitness, stabilizing the control parameters, and finding optimum solutions. (5) The CGCRA not only exhibits robust reproducibility and anti-interference to facilitate population information interaction, achieve collaborative foraging detection, and realize refined local mining, but also exhibits high consistency and reliability to circumvent dimensionality disaster, accelerate convergence efficiency, elevate solution precision, and fortify stability and robustness.

The article is structured as follows. Section 2 explores the GCRA. Section 3 elucidates the CGCRA. Section 4 delineates the simulation test and result analysis for benchmark functions. Section 5 stipulates the CGCRA for engineering designs. Section 6 summarizes the conclusion and future research.

## 2. GCRA

The greater cane rats (GCRs), which leverage specialized upper incisors to rupture sugar canes and grasses, inhabit areas adjacent to tall, vine-like grasses and water sources seen in the darkened region. White gaps and roads portray trails converging to previously identified food sources via the vine-like structures. The GCRs endure pronounced territorial awareness and instructive nighttime activity to forage and navigate the trails in reeds and grass, which eventually motivates them to find food, water resources, and refuge.

### 2.1. Population Initialization

For GCRA, the population matrix X is defined as a set of elements in the real space ${\mathbb{R}}^{n\times d}$, i.e., $X\in {\mathbb{R}}^{n\times d}$, n constitutes the population size, and d constitutes the dimensionality. The matrix X is structured as follows: rows index the GCR individuals, columns index the decision variables, and the element $x_{i,j}$ stores the corresponding position coordinate of the ith individual in the jth dimension. The matrix X is quantified as follows:

(1) $$X=\left[\begin{array}{cccc} \begin{array}{c} x_{1,1}\\ x_{2,1}\\ \vdots \\ x_{n,1} \end{array} & \begin{array}{c} x_{1,2}\\ x_{2,2}\\ \vdots \\ x_{n,2} \end{array} & \begin{array}{c} \cdots \\ \cdots \\ x_{i,j}\\ \cdots \end{array} & \begin{array}{cc} \begin{array}{c} x_{1,d-1}\\ x_{2,d-1}\\ \vdots \\ x_{n,d-1} \end{array} & \begin{array}{c} x_{1,d}\\ x_{2,d}\\ \vdots \\ x_{n,d} \end{array} \end{array} \end{array}\right]$$

The function rand is a standard random function that obeys a continuous uniform distribution over an interval [0,1], i.e., rand∼U(0,1). The function outputs a stochastic real number uniformly distributed over the interval [0,1]. The function rand constitutes the primary stochastic initialization operator to uniformly distribute individuals over the entire search space. The $UB_{j}$ constitutes the upper bound of the jth decision variable. The $LB_{j}$ constitutes the lower bound of the jth decision variable. The individual rat $x_{i,j}$ is quantified as follows:

(2) $$x_{i,j}=rand\times (UB_{j}-LB_{j})+LB_{j}$$

The dominant GCR is considered the fittest individual that specifically knows past routes to food sources and shelters; the remaining GCRs change positions according to the dominant male $x_{k}$. The factor ρ=0.5 ascertains the occurrence of the rainy season, which is implemented to equilibrate discovery and mining. The $x_{i,j}^{new}$ constitutes the refresh location of the ith individual in the jth dimension, $x_{i,j}$ constitutes the modern location of the ith individual in the jth dimension, $x_{k,j}$ constitutes the dominant male location of the kth individual in the jth dimension. The new rat $x_{i,j}^{new}$ is quantified as follows:

(3) $$x_{i,j}^{new}=0.7*\frac{\left(x_{i,j}+x_{k,j}\right)}{2}$$

### 2.2. Exploration

The GCRs manufacture the refuges of the nest and shallow burrows stretched over swamps, watersheds, and agriculture fields; they desert numerous refuges in revisiting familiar food sources along existing paths or systematically scouting new areas, identifying novel resources and establishing corresponding trails. The foraging GCRs constitute the dominant rats’ different locations. The eating GCRs constitute the obtained food sources. The dominant males retain the trail information, and the remaining GCRs utilize the data of food sources and shelters to adjust the locations. The $x_{i,j}^{new}$ constitutes the solution of the jth dimension, $x_{i,j}$ constitutes the GCR’s current position, $x_{k,j}$ constitutes the dominant male position, r constitutes the impact of food sources that promotes more exploitation, C∈[0,1] is a stochastic parameter confined within the problem space bounds to simulate the dispersed food sources and shelters, α constitutes the attenuation coefficient of food sources that force the GCRs to detect new food sources and shelters, and the coefficient β promotes GCRs to relocate to alternative accessible, ample food sources within the breeding zone. The new rat $x_{i,j}^{new}$ and forthcoming or revitalizing fresh rat $x_{i}$ are quantified as follows:

(4) $$x_{i,j}^{new}=x_{i,j}+C\times (x_{k,j}-r\times x_{i,j})$$

(5) $$x_{i}=\left\{\begin{array}{c} x_{i,j}+C\times (x_{i,j}-\alpha \times x_{k,j}),\;\;\;\;\;\;\;F_{i}^{new}<F_{i}\\ x_{i,j}+C\times (x_{m,j}-\beta \times x_{k,j}),\;\;\;\;\;\;\;\;\mathrm{otherwise} \end{array}\right.$$

The $F_{xk}$ constitutes the fitness solution of the dominant male, t constitutes the modern iteration, T constitutes the maximum iteration, μ randomly selects the values from 1 to 4, and the rand is a standard random function that obeys continuous uniform distribution over the interval [0,1], i.e., rand∼U(0,1). The control parameters r, α, and β are quantified as follows:

(6) $$r=F_{xk}-t\times (\frac{F_{xk}}{T})$$

(7) α=2×r×rand−r

(8) β=2×r×μ−r

### 2.3. Exploitation

During the breeding period and wet season, the male GCRs dissociate from the community and primarily browse in regions with plentiful food resources, which facilitates local extraction and sophisticated optimization to enrich the precision and quality of the solution. The exploitation scenario starts with the arbitrary selection female m and m≠k (dominant male). Reproduction transpires in proximity to plentiful supplies, whereas reinforcement occurs in association with chosen females. The $x_{i,j}$ constitutes the modern location of the ith individual in the jth dimension, $x_{k,j}$ constitutes the dominant male location of the kth individual in the jth dimension, $x_{m,j}$ constitutes the modern location of the mth selected female in the jth dimension, C∈[0,1] is a stochastic parameter confined within the problem space bounds to simulate the dispersed food sources and shelters, and μ randomly selects the values from 1 to 4. The new rat $x_{i,j}^{new}$ is quantified as follows:

(9) $$x_{i,j}^{new}=x_{i,j}+C\times (x_{k,j}-\mu \times x_{m,j})$$

Algorithm 1 encapsulates the pseudocode of the GCRA.
**Algorithm 1** GCRA**Begin** **Step 1.** Initialize GCR population $X_{i}(i=1,2,\ldots ,n)$ and parameter ρ
**Step 2.** Quantify the fitness of each GCR, restructure the global optimum (Gbest)              Designate the fittest GCR as dominant male $x_{k}$
             Restructure the remaining GCR based on $x_{k}$ via Equation (3) **Step 3. while**
t<T do                   **for** each GCR                     Restructure C, r, μ, α, β
                        **if**
rand<ρ                             **Exploration**                             Restructure GCR’s position via Equation (4)                          **else**                             **Exploitation**                             Restructure GCR’s position via Equation (9)                         **end if**                   **end for**                    Confirm if any solution exists outside the detect boundary and alter it                    Quantify the fitness of each GCR via a new position                     Restructure GCR’s position via Equation (5)                    Restructure Gbest and designate a new dominant male $x_{k}$                    t=t+1             **end while**
            **Return**
Gbest
**End**

## 3. CGCRA

The CGCRA generalizes traditional real-valued individual position vectors to the complex plane, which employs the algebraic properties of standard complex form to encode each search agent and enrich the representational capacity. The real and imaginary parts are decoupled into independent search dimensions, the real part accommodates the original decision variables, and the imaginary part introduces an auxiliary dimension, which facilitates secondary search operations without interfering with the primary variables. The GCRA incorporates location update, perturbation, and exploration operators to facilitate autonomous evolution and collaborative optimization and jointly construct a two-dimensional complex plane. The genetic blueprint of complex organisms is encoded in diploid or polyploid chromosomes, which perform a dimensionality-reducing translation and convert the two-dimensional encoding representation into a one-dimensional phenotypic solution domain. The CGCRA exploits two independent information dimensions to store solution space elements as the real and imaginary parts. The dual-channel encoding of the CGCRA furnishes robust spatial mapping and adaptive coordination, facilitates population collaboration and algorithmic robustness, reconciles prospecting and mining, expedites convergence efficiency, and elevates optimization precision [27,28]. For the D-dimensional problem, $R_{P}$ constitutes the CGCRA’s real part, $I_{P}$ constitutes the CGCRA’s imaginary part, $i=\sqrt{-1}$, and M constitutes the search agent scale, and the complex structure $x_{p}$ is quantified as follows:

(10) $$x_{p}=R_{p}+iI_{p},\;\;\;\;\;\;p=1,2,3,\ldots ,M$$

Table 2 delineates the chromosomes of the complex natural individual organisms.

### 3.1. Initializing CGCRA’s Population

For GCRA, the population matrix X is defined as a set of elements in the real space ${\mathbb{R}}^{n\times d}$, i.e., $X\in {\mathbb{R}}^{n\times d}$, n is the population size, and d is the dimensionality of the problem space. To expand search efficiency and foster population diversity, the CGCRA transforms the real space ${\mathbb{R}}^{n\times d}$ into the complex space ${\mathbb{C}}^{n\times d}$. The essence is to reframe the original one-dimensional real-valued search problem as a two-dimensional collaborative search over amplitude and phase. The amplitude component directly translates into the candidate solution’s positional vector within the search domain. The phase component governs the directional orientation of each individual’s search trajectory. The standardized definition of imaginary units is $i=\sqrt{-1}$. According to the definition of the complex modulus, the amplitude is defined as the Euclidean distance from the origin to a complex point, which is inherently positive definite. Consequently, this property introduces additional constraints: $\rho_{k}\geq 0,\forall k=1,2,\ldots ,M$. For the stipulated issue interval $\left[A_{k},B_{k}\right],k=1,2,\ldots ,M$, the amplitude $\rho_{k}$ and the phase $\theta_{k}$ are quantified as follows:

(11) $$\rho_{k}\in \left[0,\frac{B_{k}-A_{k}}{2}\right],\;\;\;\;\;\;k=1,2,\ldots ,M$$

(12) $$\theta_{k}=\left[-2\pi ,2\pi \right],\;\;\;\;\;\;k=1,2,\ldots ,M$$

The M constitutes the search agent scale, and $x_{Rk}$ real parts and $x_{Ik}$ imaginary parts are quantified as follows:

(13) $$x_{Rk}+ix_{Ik}=\rho_{k}(\cos\theta_{k}+i\sin\theta_{k}),\;\;\;\;\;\;k=1,2,\ldots ,M$$

### 3.2. Updating the CGCRA’s Position

The overall workflow of the CGCRA within the complex plane is structured as the hierarchical processing strategy: (1) search space mapping synchronously assigns the boundary interval of the original real-valued optimization variables to the real and imaginary parts of a complex plane, which accomplishes a transformation from the real domain to the complex plane and establishes the global search boundary. (2) The dimensional iterative search mechanism executes position perturbation, neighborhood exploitation, and global guidance of the real and imaginary parts to attain comprehensive traversal of the complex plane according to foraging and driving update rules. (3) Boundary constraint correction employs boundary truncation and rebound strategies to adjust any real or imaginary part and assure solution legitimacy and feasibility that falls outside its prescribed range. For CGCRA, $x_{(k,j)R}$ and $x_{(k,j)I}$ constitute the real and imaginary parts of the optimal dominant male GCR, and $x_{(i,j)R}$ and $x_{(i,j)I}$ constitute the real and imaginary parts of an individual GCR.

#### 3.2.1. Population Initialization

(1) Restructure the real part:

(14) $$x_{(i,j)R}^{new}=0.7*\frac{\left(x_{(i,j)R}+x_{(k,j)R}\right)}{2}$$

(2) Restructure imaginary part:

(15) $$x_{(i,j)I}^{new}=0.7*\frac{\left(x_{(i,j)I}+x_{(k,j)I}\right)}{2}$$

#### 3.2.2. Exploration

(1) Restructure the real part:

(16) $$x_{(i,j)R}^{new}=x_{(i,j)R}+C\times (x_{(k,j)R}-r\times x_{(i,j)R})$$

(17) $$x_{iR}=\left\{\begin{array}{c} x_{(i,j)R}+C\times (x_{(i,j)R}-\alpha \times x_{(k,j)R}),\;\;\;\;\;\;\;F_{iR}^{new}<F_{iR}\\ x_{(i,j)R}+C\times (x_{(m,j)R}-\beta \times x_{(k,j)R}),\;\;\;\;\;\;\;\;\mathrm{otherwise} \end{array}\right.$$

(2) Restructure imaginary part:

(18) $$x_{(i,j)I}^{new}=x_{(i,j)I}+C\times (x_{(k,j)I}-r\times x_{(i,j)I})$$

(19) $$x_{iI}=\left\{\begin{array}{c} x_{(i,j)I}+C\times (x_{(i,j)I}-\alpha \times x_{(k,j)I}),\;\;\;\;\;\;\;F_{iI}^{new}<F_{iI}\\ x_{(i,j)I}+C\times (x_{(m,j)I}-\beta \times x_{(k,j)I}),\;\;\;\;\;\;\;\;\mathrm{otherwise} \end{array}\right.$$

#### 3.2.3. Exploitation

(1) Restructure the real part:

(20) $$x_{(i,j)R}^{new}=x_{(i,j)R}+C\times (x_{(k,j)R}-\mu \times x_{(m,j)R})$$

(2) Restructure imaginary part:

(21) $$x_{(i,j)I}^{new}=x_{(i,j)I}+C\times (x_{(k,j)I}-\mu \times x_{(m,j)I})$$

### 3.3. Calculating the CGCRA’s Fitness

Rather than adopting the complex modulus as the direct objective output, the CGCRA is structured into two sequential steps: (1) decoding, the complex individual $x_{p}$ is disassembled into real part $R_{p}$ and imaginary part $I_{p}$; (2) fitness value evaluation, the CGCRA extracts the real part sequence $\left\{R_{1},R_{2},\ldots ,R_{M}\right\}$ as the final decision variable, which substitutes into the original real-valued objective function and estimates the fitness value. The real part serves as the decision variable that encapsulates the original issues, directly influences the final solution quality, and directly participates in evaluating the objective function. As an auxiliary dimension, the imaginary part actively participates in complex-plane iterations to broaden the search horizon, increase population diversity, and suppresses premature convergence, which is excluded from the objective function’s fitness evaluation without affecting solution quality assessment. The M constitutes the search agent scale, $x_{Rk}$ constitutes the real part, $x_{Ik}$ constitutes the imaginary part, $\rho_{k}$ adopts the complex modulus as the absolute magnitude of the corresponding real value, and $x_{k}$ constitutes the independent variable of the transformed real solution, the stipulated issue interval $\left[A_{k},B_{k}\right]$. The $\rho_{k}$ and $x_{k}$ are quantified as follows:

(22) $$\rho_{k}=\sqrt{x_{Rk}^{2}+x_{Ik}^{2}},\;\;\;\;\;\;k=1,2,\ldots ,M$$

(23) $$x_{k}=\rho_{k}\mathrm{sgn}\left(\sin\left(\frac{x_{Ik}}{\rho_{k}}\right)\right)+\frac{B_{k}+A_{k}}{2},\;\;\;\;\;\;k=1,2,\ldots ,M$$

### 3.4. Portraying CGCRA’s Solution Procedure

**Figure 1:**
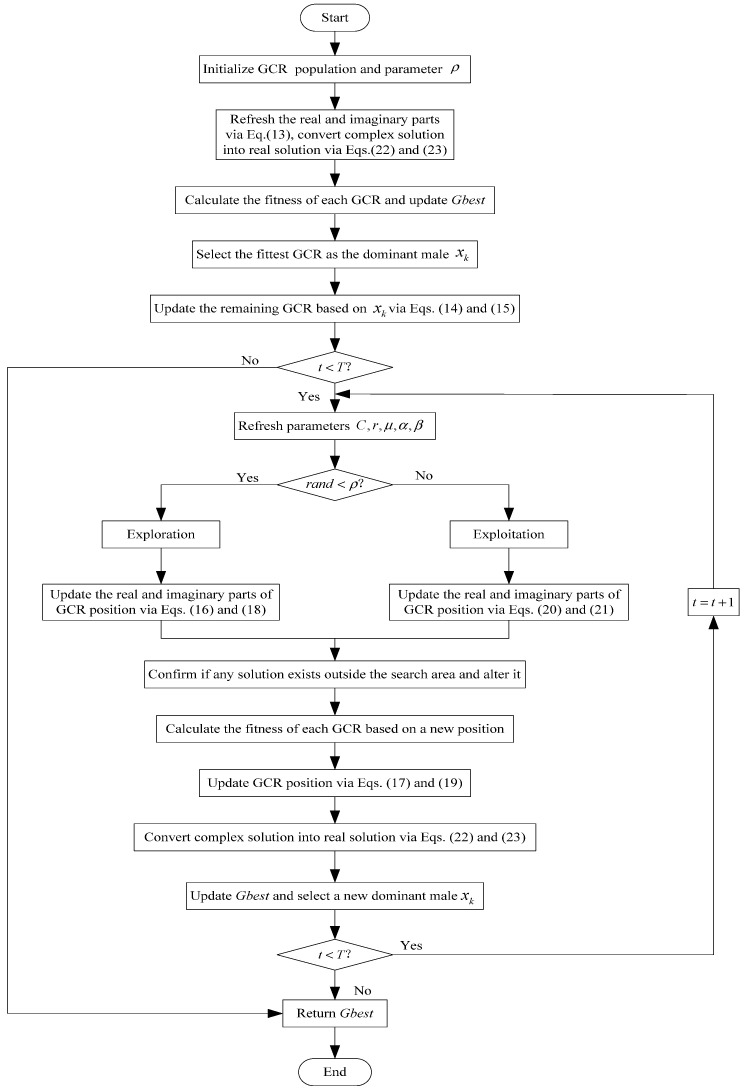
Flowchart of CGCRA.

The CGCRA endures trustworthy collaborative search attributes and versatile exploration areas to expedite convergence speed, advance calculation precision, and mitigate dimensionality disaster. Algorithm 2 encapsulates the pseudocode of the CGCRA. Figure 1 portrays the flowchart of the CGCRA.
**Algorithm 2** CGCRA**Begin** **Step 1.** Initialize GCR population $X_{i}(i=1,2,\ldots ,n)$ and parameter ρ, $\rho_{k}\in \left[0,B_{k}-A_{k}/2\right]$, $\theta_{k}=\left[-2\pi ,2\pi \right]$, restructure the real and imaginary parts via Equation (13), convert complex solution into real solution via Equations (22) and (23) **Step 2.** Quantify the fitness of each GCR, restructure the global optimum (Gbest)              Designate the fittest GCR as the dominant male $x_{k}$
             Restructure the remaining GCR based on $x_{k}$ via Equations (14) and (15) **Step 3. while**
t<T do                    **for** each GCR                        Restructure C, r, μ, α, β
                         **if**
rand<ρ
                             **Exploration**                              Restructure real and imaginary parts of position via Equations (16) and (18)                          **else**                              **Exploitation**                              Restructure real and imaginary parts of position via Equations (20) and (21)                           **end if**                    **end for**
                    Confirm if any solution exists outside the detect boundary and alter it                    Quantify the fitness of each GCR via a new position                    Restructure GCR’s position via Equations (17) and (19)                    Convert complex solutions into real solutions via Equations (22) and (23)                    Restructure Gbest and designate a new dominant male $x_{k}$                    t=t+1
           **end while**
           **Return**
Gbest
**End**

### 3.5. Complexity Analysis

Time complexity and space complexity are key indicators for measuring the computational efficiency and memory usage of the CGCRA, which is analyzed in detail as follows:

Time complexity refers to the amount of computational work required, which is used to measure the trend of the algorithm’s execution time as the input size increases. In the CGCRA, the population size is N, the maximum iteration is T, and the issue dimension is D. The CGCRA consists of initializing the population, calculating the fitness value, and updating the iterative location. For initializing the population, each GCR traverses the complex dimension and accomplishes the conversion of a real value into the complex value, which is O(N×D). For calculating the fitness value, all iterations need to quantify the GCRs’ fitness values. The temporal complexity of each GCR is dictated by the particular goal function O(f), the entire population is O(N×f), and the time complexity of assessing the fitness value at maximum iteration T is O(T×N×f). For updating the iterative position, the *D*-dimensional complex real and imaginary parts of each GCR are revised via the complex encoding mechanism and population size N. If the refreshing individual GCR’s iterative location is O(u), the refreshing of all GCRs’ iterative locations are O(N×u), and the time complexity of refreshing the iterative position at maximum iteration T is O(T×N×u). The total time complexity of the CGCRA is O(N×D+T×N×f+T×N×u), which is obtained by superimposing the time complexity of the above stages. Usually, f and u are affected by N and D, and T is a relatively large value. Therefore, the total time complexity of the CGCRA is approximated as O(T×N×D).

Space complexity describes the growth trend of the memory space occupied during execution as the input size increases. The CGCRA expands the space to store the complex-valued encoding position information of N GCR individuals with real and imaginary parts. The space occupied is closely related to the population size N, the issue dimension D, and the total space complexity of the CGCRA is O(N×D). The auxiliary variables C of the global optimal solution, individual optimal solution, and control parameters are fixed and do not change significantly, and the space complexity of auxiliary variables is O(C). Therefore, the total space complexity of the CGCRA is O(N×D+C), which is approximated as O(N×D).

## 4. Simulation Test and Result Analysis for Benchmark Functions

### 4.1. Experimental Configuration

All computational experiments were conducted on a unified hardware and software platform: a Windows 11 (64-bit) system powered by a 12th Gen Intel Core i9-12900HX 2.30 GHz CPU, complemented by 16 GB RAM, 4 TB storage, and an independent 16 GB GPU. Algorithmic implementations were carried out using MATLAB R2021b to ensure consistency across all compared methods.

### 4.2. Benchmark Functions

Table 3 delineates three categories of benchmark functions: unimodal functions $f_{1}-f_{7}$, multimodal functions $f_{8}-f_{12}$, and fixed-dimension multimodal functions $f_{13}-f_{23}$.

### 4.3. Parameter Settings

The CGCRA is compared with the BKA, EGO, HLOA, IAO, NRBO, SBOA, WSA, EHO, WO, HEOA, PO, and GCRA to demonstrate collaborative search ability and flexible exploration space. The parameter selection is outlined as follows: (1) rigorous adherence to universally recognized standards is maintained throughout the experimental framework to ensure impartiality and reproducibility. The parameters are representative empirical values that have been extensively validated in the literature, which are carefully calibrated to trade off computational cost with population heterogeneity. The universal parameters eliminate experimental interference stemming from inconsistent configurations, satisfy the scientific requirements of a fair horizontal comparison, and preclude the contrast distortion caused by parameter differentiation. (2) To satisfy the control variable requirement, we utilize the unmodified, native parameter set of the original GCRA to maintain experimental integrity. All biological behavior update formulas and the underlying evolutionary logic are inherited intact from the original algorithm. Maintaining the native parameters at fixed values is essential to guarantee that any observed improvement in algorithm performance stems solely from the complex-encoding enhancement, rather than from artificial fine-tuning of the native parameters. (3) The intrinsic mathematical mechanism of complex encoding is harnessed to ascertain the optimal value and enforce theoretical constraint satisfaction simultaneously. The performance advantage of CGCRA over comparative algorithms stems from its underlying complex dual-encoding redundancy, inherent redundancy and error tolerance, and the natural expansion of the complex-plane search space. The control parameters are based on the empirical summary values, algorithm principles, theoretical analysis, mathematical derivation, problem characteristics, or adaptive adjustment derived from the original papers. Excessive experimental simulations have confirmed the effectiveness and stability of the comparison algorithms’ parameters.

BKA [1]: aleatory numbers rand∈[0,1], r∈[0,1], constant number p=0.9, Cauchy mutation C(0,1), δ=1, μ=0.

EGO [2]: aleatory numbers $r_{1}\in [0,1]$, $r_{2}\in [0,1]$, $r_{3}\in [0,2]$, $r_{4}\in [0,100]$, a∈[0,2], coefficient vector $C_{1}\in [-a,a]$, $C_{2}\in [0,2]$.

HLOA [3]: hue circle angle h∈[0,2π], binary number σ=0   or   1, constant numbers ∂=2, $v_{0}=1$, α=π/2, $\varepsilon =1\times 10^{-6}$, g=0.009807, aleatory numbers Light∈[0,0.4046661], Dark∈[0.5440510,1], walk∈[−1,1].

IAO [4]: aleatory numbers ϑ∈[0,1], rand∈[0,1], v∈[0,1], β∈[0,1], γ∈[0,1], δ∈[0,1], ε∈[0,1], ζ∈[0,1], κ∈[0,1], w∈[0,1].

NRBO [5]: aleatory numbers rand∈(0,1), δ∈[−1,1], a∈(0,1), b∈(0,1), $r_{1}\in (0,1)$, $r_{2}\in (0,1)$, $\theta_{1}\in (-1,1)$, $\theta_{2}\in (-0.5,0.5)$, Δ∈(0,1), binary number β=0   or   1.

SBOA [6]: aleatory numbers r∈[0,1], $R_{1}\in [0,1]$, u∈[0,1], v∈[0,1], constant numbers s=0.01, η=1.5, r=0.5, arbitrary selection K=1   or   2.

WSA [7]: aleatory numbers $r_{1}\in [0,1]$, $r_{2}\in [0,1]$, $r_{3}\in [0,1]$, $r_{4}\in [0,1]$, $r_{5}\in [0,1]$, $r_{6}\in [0,1]$, step coefficient α=0.3.

EHO [8]: aleatory numbers α∈[0,1], β∈[0,2], γ∈[0,2].

WO [9]: aleatory numbers r∈[0,1], a∈[0,1], $r_{1}\in (0,1)$, $r_{2}\in (0,1)$, $r_{3}\in (0,1)$, $r_{4}\in (0,1)$, $r_{5}\in (0,1)$, P∈(0,1), θ∈(0,π).

HEOA [10]: aleatory numbers rand∈(0,1), R∈[0,1], constant numbers γ=1.5, A=0.6.

PO [11]: constant number $Seq_{Time}=1$, aleatory numbers $PF_{1}\in [0,1]$, $PF_{2}\in [0,1]$, $PF_{3}\in [0,1]$, U∈[0,1].

GCRA [12]: aleatory numbers rand∼U(0,1), μ∈[1,4], constant number ρ=0.5.

CGCRA: aleatory numbers rand∼U(0,1), μ∈[1,4], constant value ρ=0.5.

### 4.4. Simulation Test and Result Analysis

The reasonable population size facilitates the comparison approaches to investigate the solution diversity and exploit the high-quality convergence accuracy through cooperation and information exchange under limited computing resources. The maximum iteration allows the comparison algorithm to show sufficient time to implement position updates and approach the optimal solution. Each algorithm utilizes multiple independent runs to determine the different candidate solutions and comprehensively evaluate the stability and reliability. The population size is 50, the maximum iteration is 1000, and the repetition trial is 30.

Table 4 delineates the quantitative results of benchmark functions. Thirteen competing algorithms are executed on benchmark functions to systematically evaluate the CGCRA’s performance in terms of convergence efficiency, solution precision, and stability, as well as to analyze the global discovery, local utilization, and scalability with respect to problem dimensionality. The optimal value (Best), worst value (Worst), mean value (Mean), and standard deviation (Std) are adopted to comprehensively assess both convergence behavior and solution dispersion. The optimal value is to obtain the most favorable objective value among many potential solutions, which intuitively reflects the optimal effect of an algorithm in searching for a potential global optimal solution or high-quality local exploiting accuracy. The optimal value constitutes a fundamental metric for quantifying the algorithm’s proficiency in both global prospecting and local mining, which is attempted to criticize the pros and cons of numerous comparable approaches in addressing the same distinctive issue. The worst value shows the algorithm’s worst convergence accuracy, which is helpful for analyzing the stability and reliability. The gap between the worst value and the optimal value based on the initial conditions and issue characteristics reflects the fluctuation range of detection accuracy, adaptively, and anti-interference ability. The mean value constitutes the arithmetic mean of the objective functions obtained after the algorithm’s multiple runs, added and divided by the solution scale. The mean value comprehensively reflects the average quality of all candidate solutions, and the gradual optimization reflects the overall search performance to a certain extent. The discrepancy between the mean value and the optimum value is employed to estimate discovery and utilization. If the discrepancy is small, the algorithm has strong stability and convergence to determine the high-quality solution in most cases accurately. If the discrepancy is large, the algorithm has some defects of the local optimum, slow convergence speed, poor solution quality, and weak adaptability, which exhibits the improvement space to optimize the parameters and search strategy. The standard deviation is employed to characterize the dispersion degree between feasible solutions and mean values and assess the reliability and consistency. The smaller standard deviation demonstrates that feasible solutions are more densely clustered around the mean value. The algorithm has superior stability and consistency, and each operation is less affected by the initial conditions and random factors. The larger standard deviation means the distribution of feasible solutions is more dispersed, and the algorithm has poorer stability and lower repeatability. For unimodal functions $f_{1}-f_{7}$, they exhibit a global solution, continuous and smooth solution space, clear gradient direction, friendly low-dimensional testing, and a low exploration requirement, which attempts to estimate the convergence profitability and local extraction. For $f_{1}$, $f_{2}$, $f_{3}$, and $f_{4}$, the statistical quantitative indicators of the CGCRA, EGO, IAO, and GCRA retain a constant order of magnitude. The statistical metrics of the CGCRA surpass those of the BKA, HLOA, NRBO, SBOA, WSA, EHO, WO, HEOA, and PO, the CGCRA portrays trustworthy prospecting and extracting to mitigate the interference of stochastic factors, which accurately locate the exact solution in a complex distribution of locally optimal solutions. For $f_{5}$ and $f_{7}$, the statistical quantitative indicators of the CGCRA have been substantially heightened in comparison to the GCRA, the statistical metrics of the CGCRA surpass those of the BKA, EGO, HLOA, IAO, NRBO, SBOA, WSA, EHO, WO, HEOA, PO, and GCRA, and CGCRA has strong stability and a lower probability of trapping inferior solutions to determine the suboptimal solutions. For $f_{6}$, the statistical metrics of the CGCRA are inferior to that of the WSA, but surpass those of the BKA, EGO, HLOA, IAO, NRBO, SBOA, EHO, WO, HEOA, and PO, and the CGCRA exhibits fortified sustainability and repeatability. For multimodal functions $f_{8}-f_{12}$, they exhibit multiple local optima and coexist with exactly one global optimum, a complex solution space, and dimensionality sensitivity and are prone to local extreme optimality, which is utilized to verify global detection and local optima escape. For $f_{8}$ and $f_{10}$, the statistical quantitative indicators of the CGCRA, BKA, EGO, HLOA, IAO, NRBO, SBOA, WSA, WO, PO, and GCRA are all zero, and the statistical quantitative indicators of the CGCRA are superior to those of the EHO and HEOA, and the CGCRA exhibits inherent stability and minimal stochastic variability to identify the exact solution with high precision. For $f_{9}$, the statistical quantitative indicators of the CGCRA, BKA, EGO, HLOA, IAO, NRBO, SBOA, WSA, WO, HEOA, PO, and GCRA are equivalent, and the CGCRA has high practicality and repeatability. For $f_{11}$ and $f_{12}$, the statistical quantitative indicators of the CGCRA surpass those of the BKA, EGO, HLOA, IAO, NRBO, SBOA, WSA, EHO, WO, HEOA, PO, and GCRA, and the CGCRA portrays instructive reliability and adaptability in identifying suboptimal solutions. For fixed-dimension multimodal $f_{13}-f_{23}$, they exhibit multiple local extrema, intricate spatial configurations, consistent dimensionality, and favorable visualization, which is used to validate the exhaustive prospecting and extracting. For $f_{13}$, $f_{14}$, $f_{15}$, $f_{16}$, $f_{17}$, $f_{21}$, $f_{22}$, and $f_{23}$, the optimal values, worst values, and mean values of the CGCRA are equivalent, the statistical metrics of the CGCRA surpass those of the BKA, EGO, HLOA, IAO, NRBO, SBOA, WSA, EHO, WO, HEOA, PO, and GCRA, and the CGCRA encounters comprehensive adaptively and anti-interference ability to furnish a high-quality, accurate solution. The standard deviations of the CGCRA are extremely minimal, and the CGCRA maintains trustworthy consistency and repeatability in distributing realistic solutions around the mean values. For $f_{18}$, $f_{19}$, and $f_{20}$, the statistical quantitative indicators of the CGCRA dramatically strengthened in comparison to the original GCRA. The statistical quantitative indicators of the CGCRA are superior to those of the EGO, HLOA, IAO, NRBO, WSA, EHO, HEOA, and GCRA, but are inferior to those of the BKA, SBOA, WO, and PO, and the CGCRA maintains substantial dependability and adaptability to accomplish the appropriate solution. To summarize, the complex-valued encoding technique is constructed to alleviate the GCRA’s deficiencies of languid convergence efficiency, inadequate calculation precision, susceptibility to local optimum, noteworthy dimensionality disaster, weak adaptability, and a tendency towards imbalance between prospecting and mining. The CGCRA effectively integrates GCR’s multidimensional data of food resource distribution, habitat topography, water source location, and physiological characteristics and maps different environmental factors or the GCR’s behavioral pattern to the actual and imaginary parts and comprehensively characterizes the GCRS’ interactions and trade-off relationships to achieve the optimal foraging areas and trails. The CGCRA employs the complex plane to elevate the search from a one-dimensional real-valued single-point paradigm to a two-dimensional bidirectional exploration framework, which significantly enhances global exploration coverage and substantially increases the probability of converging to the global optimal solution. The CGCRA synergistically combines the original decision variables of the real part with phase-equivalent adaptive mutations of the imaginary part to induce positional offset disturbances. These disturbances facilitate escape from local traps, enable continuous traversal of neighborhood blind spots, drive iterative compression and optimization of residuals, and boost solution quality. The CGCRA employs a complex dual-coding redundant error-tolerance mechanism to enable collaborative large-scale roaming exploration, decouple the population from suboptimal initial search regions, address real-part search failures and entrapment in poor local optima, facilitate imaginary part position updates, rectify individual solutions, and avert the total collapse of search agents. The CGCRA incorporates influential flexibility and adaptability to augment population diversity and strengthen parallel computing efficiency and exhibits remarkable versatility and repeatability to prohibit search standstill and accomplish the optimum or suboptimal solution.

### 4.5. Convergence Analysis

**Figure 2:**
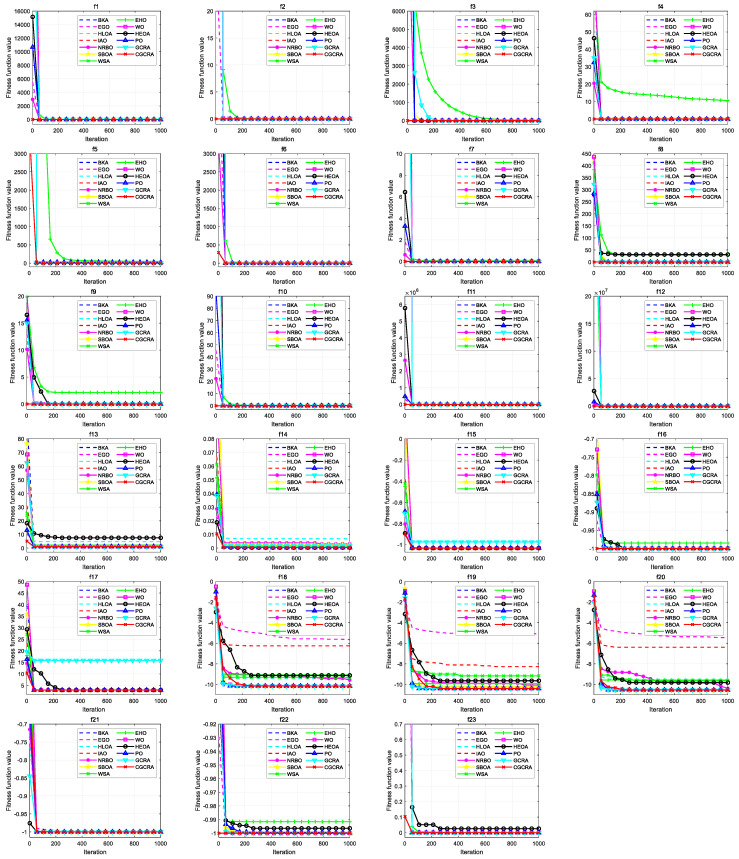
Convergence trajectories of multiple approaches.

Figure 2 portrays the convergence trajectories of multiple approaches. The convergence trajectories are utilized as a visualization tool to illustrate the evolving trend of the objective function, which receives fortified intuitiveness and sustainability for assessing the convergence rate, computational preciseness, and behavioral pattern. We can promptly ascertain if the algorithm converges to either the optimum or a near-optimal approximation by monitoring the convergence trend. If the convergence trajectories gradually decrease and tend to stabilize near a particular value, the algorithm converges to a relatively stable solution. If the convergence trajectories fluctuate violently or change irregularly, the algorithm exhibits premature convergence and slow convergence efficiency. The slope of the convergence trajectories reflects the convergence speed. If the trajectories drop rapidly in the early phase, the algorithm reflects strong detection to converge toward the optimum. If the trajectories drop slowly in the late phase, the algorithm reflects inadequate exploitation and a sluggish convergence rate. For unimodal functions $f_{1}-f_{7}$, the convergence speed and calculation precision of the CGCRA surpass those of the BKA, EGO, HLOA, IAO, NRBO, SBOA, WSA, EHO, WO, HEOA, PO, and GCRA. The CGCRA can perpetually investigate the solution zone, circumvent the curse of dimensionality, strengthen the quality of the alternatives, and distinctly harmonize with the optimum solution by leveraging the population diversity and pursuit mechanism of complex-valued encoding. For multimodal functions $f_{8}-f_{12}$, compared with the BKA, EGO, HLOA, IAO, NRBO, SBOA, WSA, EHO, WO, HEOA, PO, and GCRA, the CGCRA exhibits a smoother convergence curve, faster optimization efficiency, and higher calculation accuracy. The CGCRA not only employs the complex-valued encoding for global detection and rapid identification of the optimum solution initially but also subsequently narrows the search range for precise local exploitation and refined convergence precision. For fixed-dimensional multimodal $f_{13}-f_{23}$, the convergence speed and calculation accuracy of the CGCRA surpass those of the BKA, EGO, HLOA, IAO, NRBO, SBOA, WSA, EHO, WO, HEOA, PO, and GCRA. The CGCRA utilizes the information interaction and collaborative search mechanism between GCR individuals of the complex-valued encoding to achieve a convergence curve with less fluctuation and quickly stabilizes, which continuously explores the solution space’s potential, realizes parallel computing, avoids local optimality, improves convergence efficiency, and enhances the stability and robustness. To summarize, the CGCRA employs complex encoding with decoupled real and imaginary updates to achieve three simultaneous improvements: doubling of the equivalent population’s search cardinality, radial expansion of the initial search range on the complex plane, and elimination of the single-point start and unidirectional search drawbacks inherent to real encoding. The CGCRA retains two-stage behavioral rules: large-scale roaming exploration during the non-breeding period and localized gathering and foraging during the breeding period. The complex-plane phase rotation generates multi-directional stochastic step sizes, and facilitates natural multi-angle global exploration and rapid convergence to the globally optimal subspace. The real part implements the female’s peripheral aggregation foraging mechanism to execute small-step fine mining of extreme values within the optimal neighborhood. The imaginary part exploits complex phase variations to generate adaptive mutations, achieve fine-tuning neighborhood leak detection, pull individuals out of local optima, and sustain persistent exploration of new search domains. The CGCRA exhibits comprehensive information interaction and collaborative exploration to ascertain an accelerated convergence speed and strengthened computational precision.

### 4.6. Boxplot Analysis

**Figure 3:**
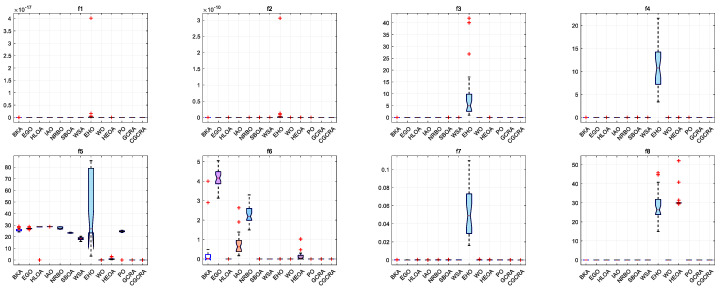

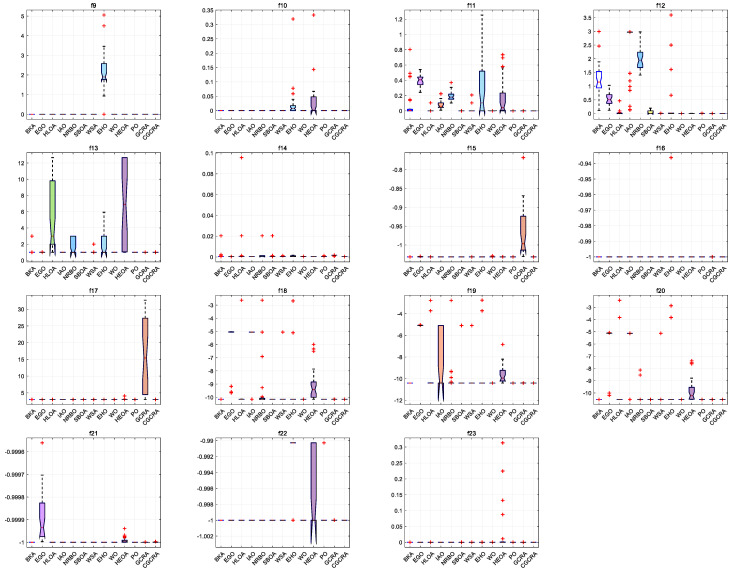
Boxplots of multiple approaches.

Figure 3 portrays boxplots of multiple approaches. The standard deviation can intuitively describe the discreteness and distribution characteristics of the data and provide visual decision support for the performance evaluation and parameter adjustment, which is a quantitative technique to evaluate the stability objectively and more accurately compare the stability variations across various approaches. If the data distribution is relatively uniform and there are no obvious outliers, the algorithm has strong consistency and reliability. Suppose many outliers or abnormal values are far from the main data distribution. In that case, the algorithm may be strongly affected by random factors or local exploration drawbacks, and further analyze the reasons. A lower standard deviation reveals that the approach portrays greater stability and delivers higher-quality solutions clustered around the mean value, which has less susceptibility to stochastic variables and initial circumstances. A larger standard deviation reveals that the approach portrays lesser stability, lower repeatability, and larger fluctuation. For unimodal functions $f_{1}-f_{7}$, the standard deviation and discreteness of the CGCRA surpass those of the BKA, EGO, HLOA, IAO, NRBO, SBOA, WSA, EHO, WO, HEOA, PO, and GCRA, the CGCRA enriches and balances the information transmission and collaborative search between GCR individuals through complex-valued operation and encoding form, which can effectively suppress the random factors’ interference, reduce the outliers’ probability, and enhance the stability and repeatability. For multimodal functions $f_{8}-f_{12}$, compared with BKA, EGO, HLOA, IAO, NRBO, SBOA, WSA, EHO, WO, HEOA, PO, and GCRA, the CGCRA has minor standard deviation and lesser discreteness, and the CGCRA has strong reliability and robustness to explore the solution space, reduce the search bias based on encoding limitation, avoid large fluctuations, and enhance the credibility and quality of the solution. For fixed-dimension multimodal $f_{13}-f_{23}$, the standard deviation and discreteness of the CGCRA surpass those of the BKA, EGO, HLOA, IAO, NRBO, SBOA, WSA, EHO, WO, HEOA, PO, and GCRA, and the CGCRA leverages the actual and imaginary parts to precisely characterize the positional distribution of the GCR individuals and furnish a more robust data foundation for assessing the detection proficiency and optimization results. To summarize, the CGCRA utilizes a complex dual-coding redundant fault-tolerant architecture that converts one-dimensional real numbers onto a two-dimensional complex plane, which achieves a low-cost doubling of population diversity, expands the search domain, enables self-adaptive mutations, and naturally decouples exploration and exploitation. When the real part fails to locate a satisfactory solution and becomes trapped in a suboptimal region, the imaginary part can independently rectify the individual’s position to mitigate the stochastic errors associated with random initialization and the random foraging step-size. The CGCRA rapidly screens high-quality individuals across the complex plane at scale, which accelerates population convergence toward the optimal region and dramatically narrows the fitness disparity between individuals. The dual-judgment mechanism reduces the influence of stochastic perturbations on the results and safeguards the algorithm against entrapment in extreme local optima. After multiple independent executions, the fitness results exhibit substantially reduced dispersion, which manifests in the variance plot as a more compact box and a diminished presence of outliers. The CGCRA portrays remarkable stability and repeatability in ascertaining a diminished standard deviation and superior discreteness.

### 4.7. Exploration and Exploitation Analysis

The fundamental principle is to maintain a dynamic balance and co-evolution between exploration and exploitation. Overemphasizing exploration may drive the algorithm into an infinite investigation, hindering convergence to the optimum solution. Overemphasizing exploitation may cause the algorithm to trigger premature convergence and lose the global optimal solution. In the early phase, the CGCRA comprehensively explores the vast solution space and discovers multiple potential high-quality solution areas in the complex plane by expanding the search range, increasing the population diversity, avoiding real domain search blind spots, and flexibly adjusting the search direction and step size. The distribution attributes of the potential solutions and the location information of the potential area for the prospective exploration region motivate the exploitation operation. If the exploration discovers that the solution distribution in a certain area is concentrated and the objective function value exhibits a downward trend, the exploitation can focus on optimizing the area and adopt a more refined search strategy, such as a smaller step size and a greater density of local search locations, to strengthen the probability of attaining the optimum solution. The exploration information can accurately guide the exploitation direction and provide the search objects. In the late stage, the CGCRA transforms potential solutions into high-quality optimal solutions by micro-adjusting the refined local search mechanism and deeply exploring the stabilized convergence. The high-quality solution obtained through exploitation is fed back to the exploration stage, which instructs the CGCRA to investigate domains associated with the existing optimum solution and advance the exploration efficiency and pertinence. If exploitation determines a high-quality solution in a local area, exploration can appropriately expand the search range around this solution to explore related potential high-quality solution areas. The exploitation drastically promotes the local regions to furnish more local feature information of the solution zone for exploration and facilitates the co-evolution of exploration and exploitation. The CGCRA has strong adaptive coordination and spatial mapping to increase information capacity and population diversity, enhance search flexibility and anti-interference ability, promote parallel computing and exploration efficiency, and facilitate spatial transformation and multi-dimensional data processing. The $median\left(x^{j}\right)$ constitutes the median of the *j*th dimension, $x_{i}^{j}$ constitutes the *j*th dimension of the *i*th search agent, N constitutes the population size, m constitutes the number of the control variables, and $Div_{j}$ constitutes the distance between the *j*th dimension of each GCR and the median of that dimension on average. The population diversity is quantified as follows:

(24) $$Div_{j}=\frac{1}{N}\sum_{i=1}^{N}\left|median\left(x^{j}\right)-x_{i}^{j}\right|$$

(25) $$Div=\frac{1}{m}\sum_{j=1}^{m}Div_{j}$$

The $Div_{\max}$ constitutes the maximum diversity. The GCRA utilizes two independent information dimensions to jointly store different elements of the solution space in the form of the real and imaginary parts, which not only has strong flexibility and adaptability to integrate GCR’s multidimensional data of the resource distribution and characterize the GCRS trade-off relationships of the optimal foraging areas and trails, but also has strong stability and repeatability in identifying accelerated convergence speed and heightened computational precision. The quantify percentage of exploration and exploitation is quantified as follows:

(26) $$Exploration\%=\left(\frac{Div}{Div_{\max}}\right)\times 100$$

(27) $$Exploitation\%=\left(\frac{\left|Div-Div_{\max}\right|}{Div_{\max}}\right)\times 100$$

### 4.8. Wilcoxon Rank-Sum Test

The Wilcoxon rank-sum test is a robust non-parametric statistical technique employed to assess whether two independent samples originate from the same underlying distribution and to quantify significant disparity between CGCRA and other approaches [29,30]. When p<0.05, the disparity is substantial. When p≥0.05, the disparity is not substantial. N/A constitutes “not applicable”. Table 5 delineates the quantitative results of the Wilcoxon rank-sum test on the benchmark functions. The CGCRA employs a dual-link parallel search strategy on the real and imaginary components, which delivers fault-tolerant redundancy, suppresses extremely poor solutions, compresses the result distribution span, maintains population heterogeneity, harmonizes exploration and exploitation, and precludes premature convergence. The CGCRA has extremely strong consistency and reliability to obtain stable sample data and realize the significant difference between the CGCRA and other algorithms.

## 5. CGCRA for Engineering Designs

To further ascertain the stability and reliability, the CGCRA is explored to address the twelve real-world engineering designs: car side impact [31], multiple disc clutch brake [32], rolling element bearing [33], gear train [34], three-bar truss [35], tubular column [36], piston lever [2], cantilevel beam [37], speed reducer [38], pressure vessel [6], tension/compression spring [39], and welden beam [4].

### 5.1. Car Side Impact

**Figure 4:**
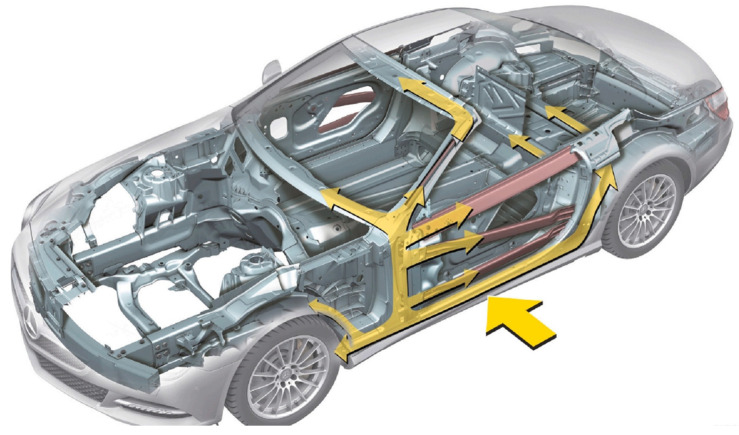
Car side impact.

The design criterion is to alleviate the vehicle’s side impact resistance and safeguard occupant protection, as portrayed in Figure 4, which involves 11 variables: B-pillar interior $x_{1}$, B-pillar fortification $x_{2}$, floor side interior $x_{3}$, transverse member $x_{4}$, door lintel $x_{5}$, door beltline fortification $x_{6}$, roof rail $x_{7}$, B-pillar interior substance $x_{8}$, floor portion interior $x_{9}$, barrier elevation $x_{10}$, and hitting location $x_{11}$. The theoretical expression is quantified as follows:

Consider

(28) $$x=[x_{1}\;\;\;x_{2}\;\;\;x_{3}\;\;\;x_{4}\;\;\;x_{5}\;\;\;x_{6}\;\;\;x_{7}\;\;\;x_{8}\;\;\;x_{9}\;\;\;x_{10}\;\;\;x_{11}]$$

Minimize

(29) $$f(x)=1.98+4.90x_{1}+6.67x_{2}+6.98x_{3}+4.01x_{4}+1.78x_{5}+2.73x_{7}$$

Subject to

(30) $$\begin{array}{l} g_{1}(x)=1.16-0.3717x_{2}x_{4}-0.00931x_{2}x_{10}-0.484x_{3}x_{9}\\ \;\;\;\;\;\;\;\;\;\;\;\;\;+0.01343x_{6}x_{10}\leq 1 \end{array}$$

(31) $$\begin{array}{l} g_{2}(x)=0.261-0.0159x_{1}x_{2}-0.188x_{1}x_{8}-0.019x_{2}x_{7}\\ \;\;\;\;\;\;\;\;\;\;\;\;\;+0.0144x_{3}x_{5}+0.0008757x_{5}x_{10}+0.080405x_{6}x_{9}\\ \;\;\;\;\;\;\;\;\;\;\;\;\;+0.00139x_{8}x_{11}+0.00001575x_{10}x_{11}\leq 0.32 \end{array}$$

(32) $$\begin{array}{l} g_{3}(x)=0.214+0.00817x_{5}-0.131x_{1}x_{8}-0.0704x_{1}x_{9}\\ \;\;\;\;\;\;\;\;\;\;\;\;\;+0.03099x_{2}x_{6}-0.018x_{2}x_{7}+0.0208x_{3}x_{8}+0.121x_{3}x_{9}\\ \;\;\;\;\;\;\;\;\;\;\;\;\;-0.00364x_{5}x_{6}+0.0007715x_{5}x_{10}-0.000535x_{6}x_{10}\\ \;\;\;\;\;\;\;\;\;\;\;\;\;+0.00121x_{8}x_{11}\leq 0.32 \end{array}$$

(33) $$\begin{array}{l} g_{4}(x)=0.074-0.061x_{2}-0.163x_{3}x_{8}+0.001232x_{3}x_{10}\\ \;\;\;\;\;\;\;\;\;\;\;\;\;-0.166x_{7}x_{9}+0.227x_{2}^{2}\leq 0.32 \end{array}$$

(34) $$\begin{array}{l} g_{5}(x)=28.98+3.818x_{3}-4.2x_{1}x_{2}+0.0207x_{5}x_{10}+6.63x_{6}x_{9}\\ \;\;\;\;\;\;\;\;\;\;\;\;\;-7.7x_{7}x_{8}+0.32x_{9}x_{10}\leq 32 \end{array}$$

(35) $$\begin{array}{l} g_{6}(x)=33.86+2.95x_{3}+0.1792x_{10}-5.057x_{1}x_{2}-11.0x_{2}x_{8}\\ \;\;\;\;\;\;\;\;\;\;\;\;\;-0.0215x_{5}x_{10}-9.98x_{7}x_{8}+22.0x_{8}x_{9}\leq 32 \end{array}$$

(36) $$g_{7}(x)=46.36-9.9x_{2}-12.9x_{1}x_{8}+0.1107x_{3}x_{10}\leq 32$$

(37) $$\begin{array}{l} g_{8}(x)=4.72-0.5x_{4}-0.19x_{2}x_{3}-0.0122x_{4}x_{10}\\ \;\;\;\;\;\;\;\;\;\;\;\;\;+0.009325x_{6}x_{10}+0.000191x_{11}^{2}\leq 4 \end{array}$$

(38) $$\begin{array}{l} g_{9}(x)=10.58-0.674x_{1}x_{2}-1.95x_{2}x_{8}+0.02054x_{3}x_{10}\\ \;\;\;\;\;\;\;\;\;\;\;\;\;-0.0198x_{4}x_{10}+0.028x_{6}x_{10}\leq 9.9 \end{array}$$

(39) $$\begin{array}{l} g_{10}(x)=16.45-0.489x_{3}x_{7}-0.843x_{5}x_{6}+0.0432x_{9}x_{10}\\ \;\;\;\;\;\;\;\;\;\;\;\;\;-0.0556x_{9}x_{11}-0.000786x_{11}^{2}\leq 15.7 \end{array}$$

Variable range

(40) $$0.5\leq x_{1}-x_{7}\leq 1.5,\;\;\;\;\;\;x_{8},x_{9}\in (0.192,0.345),\;\;\;\;\;\;-30\leq x_{10},x_{11}\leq 30$$

Table 6 delineates the quantitative results of the car side impact. The variables and weight of the CGCRA are superior to those of the other algorithms. The frequency–domain characteristics of the collision response exhibit a strong correlation with structural parameters. The CGCRA performs dual-path exploration across the complex real and imaginary components to simultaneously recognize multiple lightweight schemes, substantially constrain safety-redundant local traps, and mitigate the disruptive effect of stochastic errors. The CGCRA can expand the search space from a real-valued domain to a complex-valued domain to increase the population diversity, explore more potential candidate solutions, avoid local optimality, realize the adaptive diversity adjustment, and locate the optimum variables and weights.

### 5.2. Multiple Disc Clutch Brake

**Figure 5:**
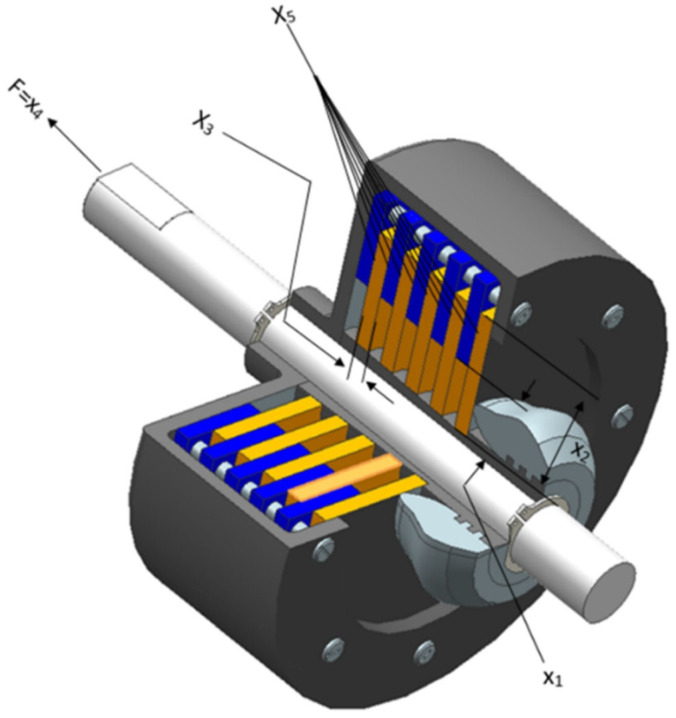
Multiple disc clutch brake.

The design criterion is to alleviate the mass of a multiple-disc clutch brake, as portrayed in Figure 5, which involves 5 variables: disc thickness t, internal radius $r_{i}$, external radius $r_{o}$, actuator exerted force F, and frictional surface size Z. The theoretical expression is quantified as follows:

Consider

(41) $$x=[x_{1}\;\;\;x_{2}\;\;\;x_{3}\;\;\;x_{4}\;\;\;x_{5}]=[r_{i}\;\;\;r_{0}\;\;\;t\;\;\;F\;\;\;Z]$$

Minimize

(42) $$f(x)=\pi t\rho (r_{0}^{2}-r_{i}^{2})(Z+1)$$

Subject to

(43) $$g_{1}(x)=r_{0}-r_{i}-\Delta r\geq 0$$

(44) $$g_{2}(x)=l_{\max}-(Z+1)(t+\delta )\geq 0$$

(45) $$g_{3}(x)=p_{\max}+p_{rz}\geq 0$$

(46) $$g_{4}(x)=p_{\max}v_{sr\;\max}-p_{rz}v_{sr}\geq 0$$

(47) $$g_{5}(x)=v_{sr\;\max}-v_{sr}\geq 0$$

(48) $$g_{6}(x)=T_{\max}-T\geq 0$$

(49) $$g_{7}(x)=M_{h}-sM_{s}\geq 0$$

(50) $$g_{8}(x)=T\geq 0$$

(51) $$M_{h}=\frac{2}{3}\mu FZ\frac{r_{0}^{3}-r_{i}^{3}}{r_{0}^{2}-r_{i}^{2}}$$

(52) $$p_{rz}=\frac{F}{\pi (r_{0}^{2}-r_{i}^{2})}$$

(53) $$v_{sr}=\frac{2\pi n(r_{0}^{3}-r_{i}^{3})}{90(r_{0}^{2}-r_{i}^{2})}$$

(54) $$T=\frac{I_{z}\pi n}{30(M_{h}+M_{f})}$$

(55) $$\Delta r=20\;\mathrm{mm},\;\;\;I_{z}=55\;\mathrm{kg}\;{\mathrm{mm}}^{2},\;\;\;p_{\max}=1\;\mathrm{Mpa},\;\;\;F_{\max}=1000\;N$$

(56) $$T_{\max}=15\;s,\;\;\;\mu =0.5,\;\;\;s=1.5,\;\;\;M_{s}=40\;\mathrm{Nm}$$

(57) $$M_{f}=3\;\mathrm{Nm},\;\;\;n=250\;\mathrm{rpm}$$

(58) $$v_{sr\;\max}=10\;\mathrm{m/s},\;\;\;l_{\max}=30\;\mathrm{mm},\;\;\;r_{i\;\;\min}=60$$

(59) $$r_{i\;\max}=80,\;\;\;r_{o\;\min}=90$$

(60) $$r_{o\;\max}=110,\;\;\;t_{\min}=1.5,\;\;\;t_{\max}=3,\;\;\;F_{\min}=600$$

(61) $$F_{\max}=1000,\;\;\;Z_{\min}=2,\;\;\;Z_{\max}=9$$

Table 7 delineates the quantitative results of the multiple-disc clutch brake. The CGCRA incorporates the complex-value encoding’s refresh rules and operation characteristics to recognize the solution domain and extract the optimum resolution. The algorithm maps integer decision variables to the phase angles of the complex space, leveraging quadrant partitions to encode discrete value ranges and the amplitude to represent continuous variables. The complex-valued encoding method retains algorithmic differentiability and eliminates the associated degradation in search efficiency. The CGCRA leverages a flexible domain and search mechanism to immediately accomplish the optimum solution and swiftly execute cross-region searches, exhibiting substantial trustworthiness and dependability in acquiring superior variables and weight.

### 5.3. Rolling Element Bearing

**Figure 6:**
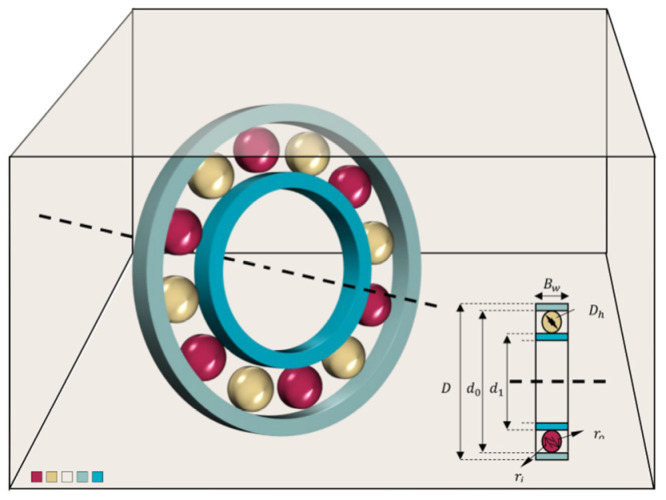
Rolling element bearing.

The design criterion is to bolster the dynamic load-carrying capacity of a rolling element bearing, as portrayed in Figure 6, which involves 10 variables: pitch diameter $D_{m}$, ball diameter $D_{b}$, ball size Z, interior $f_{i}$, exterior $f_{o}$, and racetrack distortion coefficients $K_{D\min}$, $K_{D\max}$, ε, e, and ζ. The theoretical expression is quantified as follows:

Consider

(62) $$\begin{array}{l} x=[x_{1}\;\;\;x_{2}\;\;\;x_{3}\;\;\;x_{4}\;\;\;x_{5}\;\;\;x_{6}\;\;\;x_{7}\;\;\;x_{8}\;\;\;x_{9}\;\;\;x_{10}]\\ \;\;=[D_{m}\;\;\;\;D_{b}\;\;\;Z\;\;\;f_{i}\;\;\;f_{o}\;\;\;K_{D\min}\;\;\;K_{D\max}\;\;\;\varepsilon \;\;\;e\;\;\;\zeta ] \end{array}$$

Maximize

(63) $$C_{d}=\left\{\begin{array}{c} f_{c}Z^{2/3}D_{b}^{1.8},\;\;\;\;\;\;\;\;\;\;\;\;\;if\;\;D\leq 25.4\;\mathrm{mm}\\ 3.647f_{c}Z^{2/3}D_{b}^{1.4},\;\;\;\;\;\;\;if\;\;\;D>25.4\;\mathrm{mm} \end{array}\right.$$

Subject to

(64) $$g_{1}(x)=\frac{\phi_{0}}{2\sin^{-1}(D_{b}/D_{m})}-Z+1\leq 0$$

(65) $$g_{2}(x)=2D_{b}-K_{D\min}(D-d)\geq 0$$

(66) $$g_{3}(x)=K_{D\max}(D-d)-2D_{b}\geq 0$$

(67) $$g_{4}(x)=\zeta B_{\omega }-D_{b}\leq 0$$

(68) $$g_{5}(x)=D_{m}-0.5(D+d)\geq 0$$

(69) $$g_{6}(x)=(0.5+e)(D+d)-D_{m}\geq 0$$

(70) $$g_{7}(x)=0.5(D-D_{m}-D_{b})-\varepsilon D_{b}\geq 0$$

(71) $$g_{8}(x)=f_{i}\geq 0.515$$

(72) $$g_{9}(x)=f_{o}\geq 0.515$$

(73) fc=37.911+1.041−r1+r1.72fi(2fo−1)fo(2fi−1)0.41103−0.3                     ×r0.3(1−r)1.39(1+r)1/32fi2fi−10.41

(74) $$x={\left\{\frac{\left(D-d\right)}{2}-3\frac{T}{4}\right\}}^{2}+{\left\{\frac{D}{2}-\frac{T}{4}-D_{b}\right\}}^{2}-{\left\{\frac{d}{2}+\frac{T}{4}\right\}}^{2}$$

(75) $$y=2\left\{\frac{\left(D-d\right)}{2}-3\frac{T}{4}\right\}\left\{\frac{D}{2}-\frac{T}{4}-D_{b}\right\}$$

(76) $$\phi_{o}=2\pi -2\cos^{-1}\left(\frac{x}{y}\right)$$

(77) $$r=\frac{D_{b}}{D_{m}},\;\;\;f_{i}=\frac{r_{i}}{D_{b}},\;\;\;f_{o}=\frac{r_{o}}{D_{b}},\;\;\;T=D-d-2D_{b}$$

(78) $$D=160,\;\;\;d=90,\;\;\;B_{\omega }=30,\;\;\;r_{i}=r_{o}=11.033$$

Variable range

(79) $$0.5(D+d)\leq D_{m}\leq 0.6(D+d)$$

(80) $$0.15(D-d)\leq D_{b}\leq 0.45(D-d)$$

(81) $$4\leq Z\leq 50,\;\;\;0.515\leq f_{i},f_{o}\leq 0.6$$

(82) $$0.4\leq K_{D\min}\leq 0.5,\;\;\;0.6\leq K_{D\min}\leq 0.7$$

(83) 0.3≤ε≤0.4,   0.02≤e≤0.1,   0.6≤ζ≤0.85

Table 8 delineates the quantitative results of the rolling element bearing. The CGCRA utilizes exploration and exploitation to achieve information interaction and cooperation to explore the superior variables and costs. The CGCRA harnesses the imaginary part to conduct global large-scale exploration, traverse the entire ranges of both the pitch circle diameter and the rolling element diameter, and lock onto intervals with superior load-carrying potential. The CGCRA leverages the real part to materialize local refinement, fine-tuning the curvature coefficient and rolling element count within high-quality intervals, and achieve an accurate approximation of the maximum load. Exploration provides rich initialization information and a wide search domain for exploitation. Exploitation provides deep precision mining and local refinement optimization for prospecting. The CGCRA has bettered the solution’s quality and convergence consistency.

### 5.4. Gear Train

**Figure 7:**
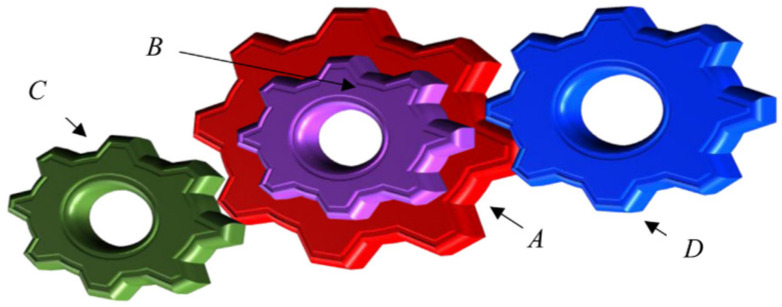
Gear train.

The design criterion is to alleviate the cost of a gear ratio, as portrayed in Figure 7, which involves 4 variables: tooth size attributes of four distinct gearwheels $n_{A}$, $n_{B}$, $n_{C}$, and $n_{D}$. The theoretical expression is quantified as follows:

Consider

(84) $$x=[x_{1}\;\;\;x_{2}\;\;\;x_{3}\;\;\;x_{4}]=[n_{A}\;\;\;n_{B}\;\;\;n_{C}\;\;\;n_{D}]$$

Minimize

(85) $$f(x)={\left(\frac{1}{6.931}-\frac{x_{3}x_{2}}{x_{1}x_{4}}\right)}^{2}$$

Variable range

(86) $$12\leq x_{i}\leq 60,\;\;\;i=1,2,\ldots ,4$$

Table 9 delineates the quantitative results of the gear train. The CGCRA can determine more suitable variable combinations and quickly approach the exact solution through the complex-valued encoding’s flexibility and the CGCRA’s group intelligent search strategy. The CGCRA employs the imaginary part to execute a wide-ranging global detection, optimize continuous variables of the modulus, tooth width, and displacement coefficient, and identify lightweight construction, high-quality parameter domains. The CGCRA manipulates the real part to actualize local fine-grained mining, process the discrete integer tooth-number variable, and approximate the optimal constraint boundary. The CGCRA has good openness and scalability to achieve the different modes’ global detection and each mode’s local exploitation. The CGCRA exhibits instructive superiority and consistency in attaining the global solution.

### 5.5. Three-Bar Truss

**Figure 8:**
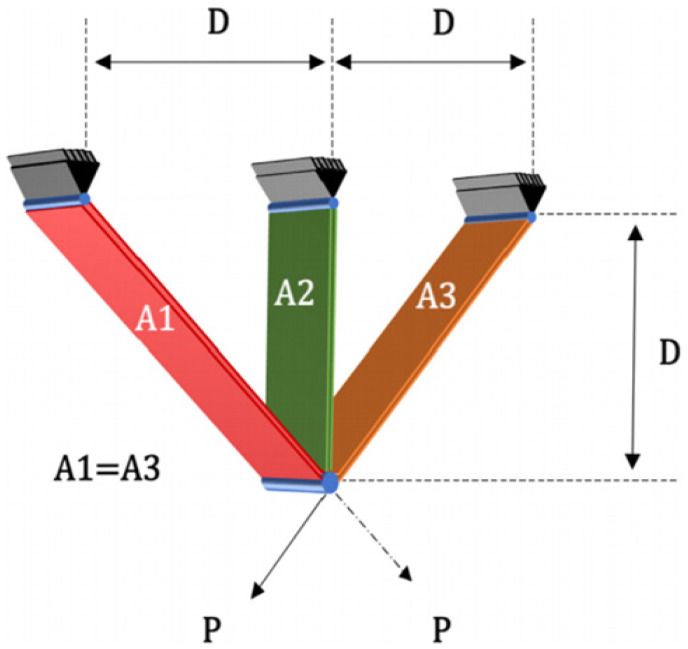
Three-bar truss.

The design criterion is to alleviate the structure weight, as portrayed in Figure 8, which involves 2 variables: transverse sections $A_{1}$ and $A_{2}$. The theoretical expression is quantified as follows:

Consider

(87) $$x=[x_{1}\;\;\;x_{2}]=[A_{1}\;\;\;A_{2}]$$

Minimize

(88) $$f(x)=(2\sqrt{2}x_{1}+x_{2})\times l$$

Subject to

(89) $$g_{1}(x)=\frac{\sqrt{2}x_{1}+x_{2}}{\sqrt{2}x_{1}^{2}+2x_{1}x_{2}}P-\sigma \leq 0$$

(90) $$g_{2}(x)=\frac{x_{2}}{\sqrt{2}x_{2}+2x_{1}x_{2}}P-\sigma \leq 0$$

(91) $$g_{3}(x)=\frac{1}{\sqrt{2}x_{2}+x_{1}}P-\sigma \leq 0$$

(92) $$l=100\;\mathrm{cm},\;\;\;\;\;\;P=2\;\mathrm{kN}/{\mathrm{cm}}^{2},\;\;\;\;\;\;\sigma =2\;\mathrm{kN}/{\mathrm{cm}}^{2}$$

Variable range

(93) $$0\leq x_{1},x_{2}\leq 1$$

Table 10 delineates the quantitative results of a three-bar truss. The CGCRA implements a hierarchical constraint strategy: the real part enforces stress and displacement limits with strict adherence, and the imaginary part focuses on lightweight optimization precisely at the boundaries of the feasible constraint region. The CGCRA employs amplitude clipping to rectify individuals that exceed the search boundaries and retain potentially high-quality solutions. The CGCRA incorporates the sophisticated foraging behaviors of the GCRs and unique search advantages of the complex-value encoding to furnish the flexible collaborative scouring, cover the search dimension and domain of the solution zone, circumvent local optima, and strengthen the pursuit efficiency and precision. The CGCRA exhibits substantial convergence and consistency in identifying the optimal solution.

### 5.6. Tubular Column

**Figure 9:**
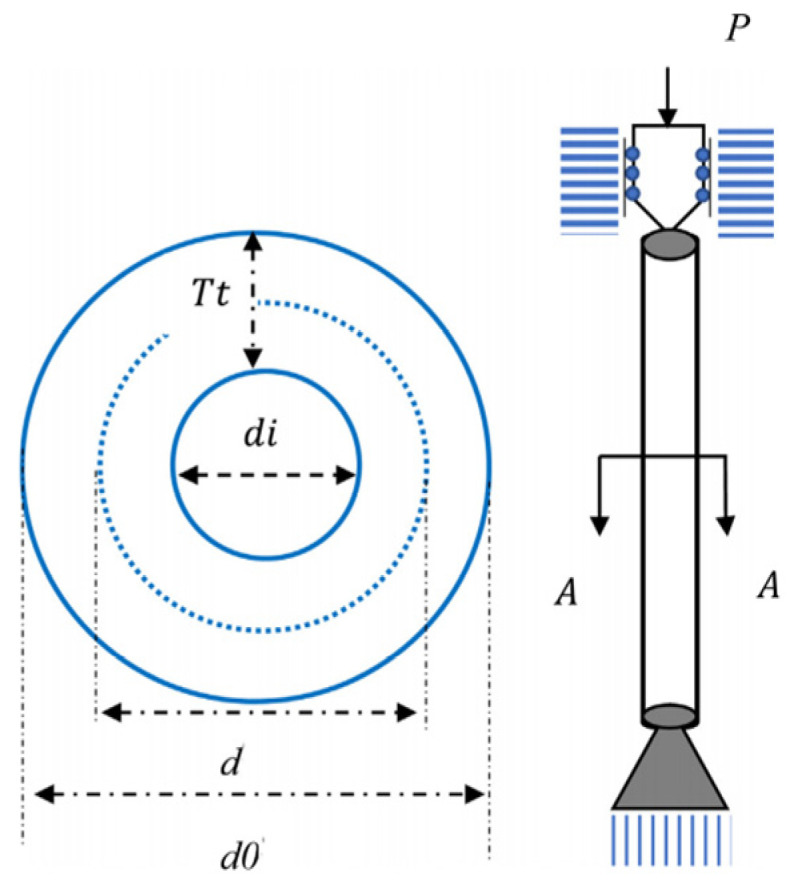
Tubular column.

The design criterion is to prioritize cost reduction and withstand compressive load-bearing capability, as portrayed in Figure 9, which involves two variables: average diameter d and average thickness t. The theoretical expression is quantified as follows:

Consider

(94) $$x=[x_{1}\;\;\;x_{2}]=[d\;\;\;t]$$

Minimize

(95) $$f(x)=9.82x_{1}x_{2}+2x_{1}$$

Subject to

(96) $$g_{1}(x)=\frac{P}{\pi x_{1}x_{2}\sigma_{y}}-1\leq 0$$

(97) $$g_{2}(x)=\frac{8PL^{2}}{\pi^{3}Ex_{1}x_{2}(x_{1}^{2}+x_{2}^{2})}-1\leq 0$$

(98) $$g_{3}(x)=\frac{2.0}{x_{1}}-1\leq 0$$

(99) $$g_{4}(x)=\frac{x_{1}}{14}-1\leq 0$$

(100) $$g_{5}(x)=\frac{0.2}{x_{2}-1}\leq 0$$

(101) $$g_{6}(x)=\frac{x_{2}}{0.8}-1\leq 0$$

(102) $$\sigma_{y}=500\;\mathrm{kgf}/{\mathrm{cm}}^{2},\;\;\;\;\;\;E=0.85\times 10^{6}\;\mathrm{kgf}/{\mathrm{cm}}^{2},\;\;\;\;\;\;P=2500\;\mathrm{kgf},\;\;\;\;\;\;L=250\;\mathrm{cm}$$

Variable range

(103) $$2\leq x_{1}\leq 14,\;\;\;\;\;\;0.2\leq x_{2}\leq 0.8$$

Table 11 delineates the quantitative results of the tubular column. By mapping two distinct inner-diameter and wall-thickness combinations onto the real and imaginary parts, the CGCRA effectively searches twice as many individuals as conventional methods for the same population size. This method simultaneously mitigates premature convergence and reduces the computational burden associated with repeated buckling and stress checks. The real part prioritizes constraint satisfaction of buckling and strength, and the imaginary part is dedicated to lightweight optimization along the constraint boundaries. The CGCRA integrates multi-dimensional data of food resource distribution, habitat topography, water source location, and physiological characteristics to increase information capacity and population diversity, enhance search flexibility and anti-interference ability, promote parallel computing and exploration efficiency, and facilitate spatial transformation and multi-dimensional data processing.

### 5.7. Piston Lever

**Figure 10:**
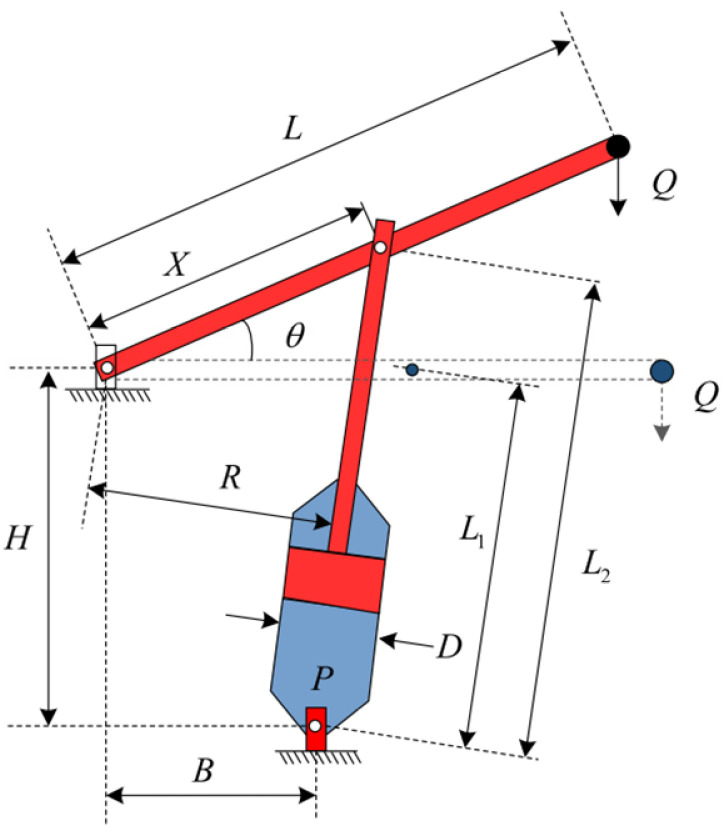
Piston lever.

The design criterion is to alleviate the oil displacement resulting from piston elevation over the interval $\left[0^{{}^{\circ}},45^{{}^{\circ}}\right]$, as portrayed in Figure 10, which involves 4 variables: piston components H, B, X, and D. The theoretical expression is quantified as follows:

Consider

(104) $$x=[x_{1}\;\;\;x_{2}\;\;\;x_{3}\;\;\;x_{4}]=[H\;\;\;B\;\;\;D\;\;\;X]$$

Minimize

(105) $$f(x)=\frac{1}{4}\pi x_{3}^{2}(L_{2}-L_{1})$$

Subject to

(106) $$g_{1}(x)=QL\cos\theta -RF\leq 0$$

(107) $$g_{2}(x)=Q(L-x_{4})-M_{\max}\leq 0$$

(108) $$g_{3}(x)=\frac{6}{5}\times (L_{2}-L_{1})-L_{1}\leq 0$$

(109) $$g_{4}(x)=\frac{x_{3}}{2}-x_{2}\leq 0$$

(110) $$R=\frac{\left|-x_{4}(x_{4}\sin\theta +x_{1})+x_{1}(x_{2}-x_{4}\cos\theta )\right|}{\sqrt{{\left(x_{4}-x_{2}\right)}^{2}+x_{1}^{2}}}$$

(111) $$F=\frac{\pi Px_{3}^{2}}{4}$$

(112) $$L_{1}=\sqrt{{\left(x_{4}-x_{2}\right)}^{2}+x_{1}^{2}}$$

(113) $$L_{2}=\sqrt{{\left(x_{4}\sin\theta +x_{1}\right)}^{2}+{\left(x_{2}-x_{4}\cos\theta \right)}^{2}}$$

(114) $$\theta =45^{{}^{\circ}},\;\;\;Q=10000\;\mathrm{lbs},\;\;\;L=240\;\mathrm{in},\;\;\;M_{\max}=1.8\times 10^{6}\;\mathrm{lbs}\;\;\mathrm{in},\;\;\;P=1500\;\mathrm{psi}$$

Variable range

(115) $$0.05\leq x_{1},x_{2},x_{4}\leq 500,\;\;\;\;\;\;0.05\leq x_{3}\leq 120$$

Table 12 delineates the quantitative results of the piston lever. The CGCRA employs a dual-dimensional search over the amplitude and phase of the complex space, which simultaneously enriches the solution set, counteracts population homogenization, mitigates premature convergence, reduces buckling capacity, and approximates the feasible region of the optimal solution under coarse constraints. The CGCRA has strong operability and practicality to achieve the rapid positioning of the global detection area and the refined search of the local exploitation area. The CGCRA utilizes the collaborative search of multiple individuals to reduce the uneven distribution of initial solutions and approach the optimum solution. The CGCRA can effectively deal with the complexity and uncertainty of the issue during the search process, which possesses exceptional flexibility and adjustability to facilitate high-quality variables and solutions.

### 5.8. Cantilever Beam

**Figure 11:**
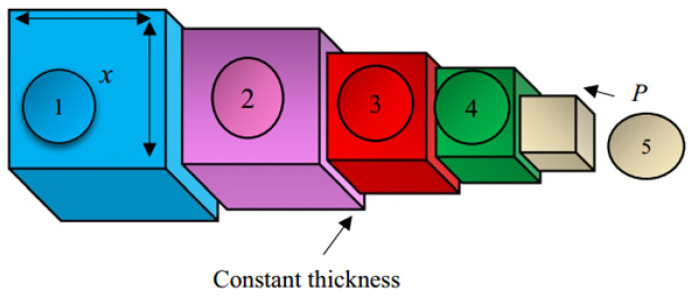
Cantilever beam.

The design criterion is to prioritize weight minimization of a rectangular-section cantilever beam, as portrayed in Figure 11, which involves five variables: height or widths of the square blocks $x_{1}$, $x_{2}$, $x_{3}$, $x_{4}$, and $x_{5}$. The theoretical expression is quantified as follows:

Consider

(116) $$x=[x_{1}\;\;\;x_{2}\;\;\;x_{3}\;\;\;x_{4}\;\;\;\;x_{5}]$$

Minimize

(117) $$f(x)=0.6224(x_{1}+x_{2}+x_{3}+x_{4}+x_{5})$$

Subject to

(118) $$g(x)=\frac{61}{x_{1}^{3}}+\frac{37}{x_{2}^{3}}+\frac{19}{x_{3}^{3}}+\frac{7}{x_{4}^{3}}+\frac{1}{x_{5}^{3}}\leq 1$$

Variable range

(119) $$0.01\leq x_{1},x_{2},x_{3},x_{4},x_{5}\leq 100$$

Table 13 delineates the quantitative results of the cantilever beam. The CGCRA decomposes a conventional single-dimensional encoding into two independent real and imaginary components. The transformation preserves individuals that meet deflection and stress constraints without requiring any increase in the encoding length. The CGCRA increases the population’s information density, enables rapid coarse-feasibility screening, and elevates the probability of locating a globally feasible solution. The optimum variables and weight of the CGCRA are superior to those of the other algorithms. The CGCRA not only retains fortified spatial mapping and adaptive coordination to facilitate population collaboration and strengthen the foraging efficiency, but also exhibits substantial consistency and repeatability to preserve population diversity, restrict local convergence, foster thorough exploration of the solution zone, expedite the information collaboration, and accomplish the high-quality global solution.

### 5.9. Speed Reducer

**Figure 12:**
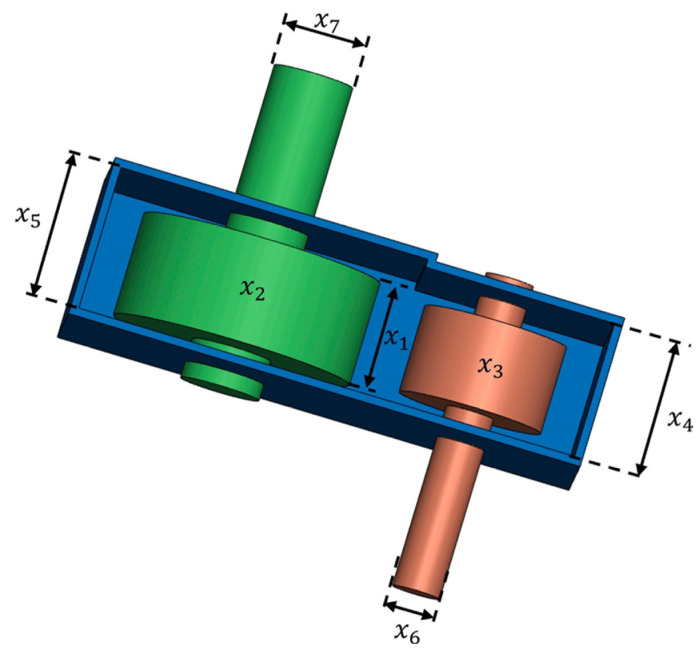
Speed reducer.

The design criterion is to prioritize weight minimization, as portrayed in Figure 12, which involves seven variables: facial breadth b, gear assembly m, pinion teeth z, first shaft span $l_{1}$, second shaft span $l_{2}$, first shaft diameter $d_{1}$, and second shaft diameter $d_{2}$. The theoretical expression is quantified as follows:

Consider

(120) $$x=[x_{1}\;\;\;x_{2}\;\;\;x_{3}\;\;\;x_{4}\;\;\;\;x_{5}\;\;\;\;x_{6}\;\;\;\;x_{7}]=[b\;\;\;m\;\;\;z\;\;\;\;l_{1}\;\;\;l_{2}\;\;\;d_{1}\;\;\;d_{2}]$$

Minimize

(121) $$\begin{array}{l} f(x)=0.7854x_{1}x_{2}^{2}(3.3333x_{3}^{2}+14.9334x_{3}-43.0934)\\ \;\;\;\;\;\;\;\;\;\;-1.508x_{1}(x_{6}^{2}+x_{7}^{2})+7.4777(x_{6}^{3}+x_{7}^{3})+0.7854(x_{4}x_{6}^{2}+x_{5}x_{7}^{2}) \end{array}$$

Subject to

(122) $$g_{1}(x)=\frac{27}{x_{1}x_{2}^{2}x_{3}^{2}}-1\leq 0$$

(123) $$g_{2}(x)=\frac{397.5}{x_{1}x_{2}^{2}x_{3}}-1\leq 0$$

(124) $$g_{3}(x)=\frac{1.93x_{4}^{3}}{x_{2}x_{6}^{4}x_{3}}-1\leq 0$$

(125) $$g_{4}(x)=\frac{1.93x_{5}^{3}}{x_{2}x_{7}^{5}x_{3}}-1\leq 0$$

(126) $$g_{5}(x)=\frac{[(745x_{4}/x_{2}x_{3}{)}^{2}+16.9\times 10^{6}{]}^{1/2}}{110x_{6}^{3}}-1\leq 0$$

(127) $$g_{6}(x)=\frac{[(745x_{5}/x_{2}x_{3}{)}^{2}+157.5\times 10^{6}{]}^{1/2}}{85x_{7}^{3}}-1\leq 0$$

(128) $$g_{7}(x)=\frac{x_{2}x_{3}}{40}-1\leq 0$$

(129) $$g_{8}(x)=\frac{5x_{2}}{x_{1}}-1\leq 0$$

(130) $$g_{9}(x)=\frac{x_{1}}{12x_{2}}-1\leq 0$$

(131) $$g_{10}(x)=\frac{1.5x_{6}+1.9}{x_{4}}-1\leq 0$$

(132) $$g_{11}(x)=\frac{1.1x_{7}+1.7}{x_{5}}-1\leq 0$$

Variable range

(133) $$2.6\leq x_{1}\leq 3.6,\;\;\;0.7\leq x_{2}\leq 0.8,\;\;\;17\leq x_{3}\leq 28,\;\;\;7.3\leq x_{4},x_{5}\leq 8.3,\;\;\;2.9\leq x_{6}\leq 3.9,\;\;\;5.0\leq x_{7}\leq 5.5$$

Table 14 delineates the quantitative results of the speed reducer. The CGCRA employs the imaginary part to conduct a global exploration that exhaustively traverses the entire domain of continuous design variables over a broad range, converges on coarse feasible intervals, and provides a strong foundation for subsequent local refinement. The CGCRA adopts the real part local refinement, fine-tuning the discrete tooth number and structural dimensions to drive the design toward the minimum weight located precisely on the constraint boundary. The CGCRA utilizes the complex-valued search diversity and initialization randomness to detect the global solution space, explore a local optimum solution, avoid local optimality, and enrich search trajectories, which combines the cooperation and information sharing mechanism of the CGCRA to search with different step sizes and directions, accelerate the convergence efficiency, and achieve the optimal solution.

### 5.10. Pressure Vessel

**Figure 13:**
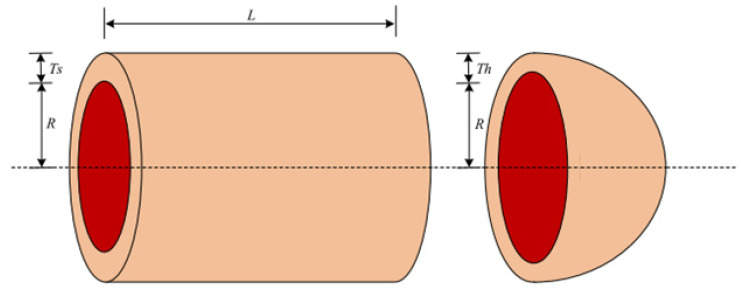
Pressure vessel.

The design criterion is to prioritize the reduction of construction cost, as portrayed in Figure 13, which involves four variables: shell thickness $T_{s}$, internal radius R, cranial thickness $T_{h}$, and cranial length L. The theoretical expression is quantified as follows:

Consider

(134) $$x=[x_{1}\;\;\;x_{2}\;\;\;x_{3}\;\;\;x_{4}\;]=[T_{s}\;\;\;T_{h}\;\;\;R\;\;\;L]$$

Minimize

(135) $$f(x)=0.6224x_{1}x_{3}x_{4}+1.7781x_{2}x_{3}^{2}+3.1661x_{1}^{2}x_{4}+19.84x_{1}^{2}x_{3}$$

Subject to

(136) $$g_{1}(x)=-x_{1}+0.0193x_{3}\leq 0$$

(137) $$g_{2}(x)=-x_{2}+0.00954x_{3}\leq 0$$

(138) $$g_{3}(x)=-\pi x_{3}^{2}x_{4}-\frac{4}{3}\pi x_{3}^{3}+1296000\leq 0$$

(139) $$g_{4}(x)=x_{4}-240\leq 0$$

Variable range

(140) $$0\leq x_{1},x_{2}\leq 99,\;\;\;\;\;\;10\leq x_{3},x_{4}\leq 200$$

Table 15 delineates the quantitative results of the pressure vessel. The CGCRA is founded on a complex-plane diploid bivariate architecture to decouple real and imaginary components and achieve parallel exploration and exploitation, spatial expansion, scale adaptation, and soft boundary mapping. This encoding strategy is intrinsically aligned with the engineering characteristics of the pressure cylinder at the coding level, ultimately converging to a structural optimization that achieves lower cost and higher reliability. The CGCRA has strong consistency and repeatability to explore the solution space, increase the spatial dimension, achieve a refined search, and enhance robustness and reliability. The CGCRA not only harnesses population diversity and search flexibility to swiftly localize high-quality solution subspaces and substantially boost global optimum convergence, but also employs the ameliorated regional searches to deliver a broader array of candidate solutions and acquire the optimum solution.

### 5.11. Tension/Compression Spring

**Figure 14:**
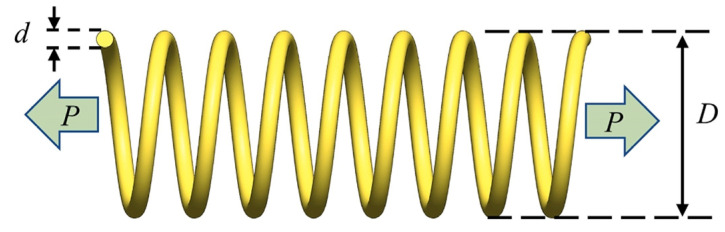
Tension/compression spring.

The design criterion is to prioritize weight minimization, as portrayed in Figure 14, which involves three variables: wire thickness d, coil diameter D, and coil size N. The theoretical expression is quantified as follows:

Consider

(141) $$x=[x_{1}\;\;\;x_{2}\;\;\;x_{3}\;]=[d\;\;\;D\;\;\;N]\;\;\;$$

Minimize

(142) $$f(x)=(x_{3}+2)x_{2}x_{1}^{2}$$

Subject to

(143) $$g_{1}(x)=1-\frac{x_{2}^{3}x_{3}}{71785x_{1}^{4}}\leq 0$$

(144) $$g_{2}(x)=\frac{4x_{2}^{2}-x_{1}x_{2}}{12566(x_{2}x_{1}^{3}-x_{1}^{4})}+\frac{1}{5108x_{1}^{2}}\leq 0$$

(145) $$g_{3}(x)=1-\frac{140.45x_{1}}{x_{2}^{2}x_{3}}\leq 0$$

(146) $$g_{4}(x)=\frac{x_{1}+x_{2}}{1.5}-1\leq 0$$

Variable range

(147) $$0.05\leq x_{1}\leq 2,\;\;\;\;\;\;0.25\leq x_{2}\leq 1.3,\;\;\;\;\;\;2\leq x_{3}\leq 15$$

Table 16 delineates the quantitative results of the tension/compression spring. The optimum variables and solutions of the CGCRA are superior to those of the other approaches. The CGCRA utilizes the real part for local fine-grained exploitation, meticulously tuning control parameters to achieve significant weight reduction. The imaginary part facilitates a continuous exploration process that continually replenishes and updates search agents. This mechanism is particularly effective in avoiding premature convergence in problems with multiple extrema, the algorithm might otherwise become trapped in suboptimal regions. The CGCRA comprehensively investigates the extensive solution zone and identifies multiple potential high-quality solution areas in the complex plane by expanding the search range, increasing population diversity, avoiding real domain search blind spots, and dynamically modulating the search orientation and step length.

### 5.12. Welden Beam

**Figure 15:**
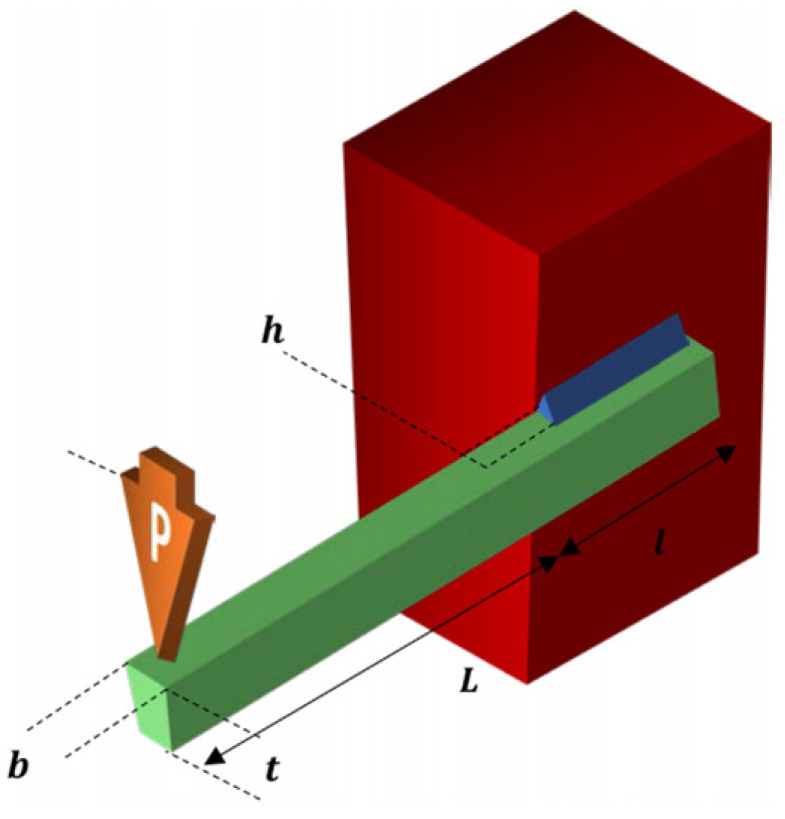
Welden beam.

The design criterion is to prioritize the reduction of construction cost, as portrayed in Figure 15, which involves four variables: thickness h, length l, height t, and width b. The theoretical expression is quantified as follows:

Consider

(148) $$x=[x_{1}\;\;\;x_{2}\;\;\;x_{3}\;\;\;x_{4}]=[h\;\;\;l\;\;\;t\;\;\;b]$$

Minimize

(149) $$f(x)=1.10471x_{1}^{2}x_{2}+0.04811x_{3}x_{4}(14.0+x_{2})$$

Subject to

(150) $$g_{1}(x)=\tau (x)-\tau_{\max}\leq 0$$

(151) $$g_{2}(x)=\sigma (x)-\sigma_{\max}\leq 0$$

(152) $$g_{3}(x)=\delta (x)-\delta_{\max}\leq 0$$

(153) $$g_{4}(x)=x_{1}-x_{4}\leq 0$$

(154) $$g_{5}(x)=P-P_{c}(x)\leq 0$$

(155) $$g_{6}(x)=0.125-x_{1}\leq 0$$

(156) $$g_{7}(x)=1.10471x_{1}^{2}+0.04811x_{3}x_{4}(14.0+x_{2})-5.0\leq 0$$

(157) $$\tau (x)=\sqrt{{\left(\tau^{\prime }\right)}^{2}+2\tau^{\prime }\tau^{\prime \prime }\frac{x_{2}}{2R}+{\left(\tau^{\prime \prime }\right)}^{2}}$$

(158) $$\tau^{\prime }=\frac{P}{\sqrt{2}x_{1}x_{2}},\;\;\;\;\;\;\tau^{\prime \prime }=\frac{MP}{J},\;\;\;\;\;\;M=P(L+\frac{x_{2}}{2})$$

(159) $$R=\sqrt{\frac{x_{2}^{2}}{4}+{\left(\frac{x_{1}+x_{3}}{2}\right)}^{2}}$$

(160) $$J=2\left\{\sqrt{2}x_{1}x_{2}\left[\frac{x_{2}^{2}}{4}+{\left(\frac{x_{1}+x_{3}}{2}\right)}^{2}\right]\right\}$$

(161) $$\sigma (x)=\frac{6PL}{x_{4}x_{3}^{2}},\;\;\;\;\;\;\delta (x)=\frac{6PL^{3}}{Ex_{3}^{2}x_{4}}$$

(162) $$P_{c}(x)=\frac{4.103E\sqrt{\frac{x_{3}^{2}x_{4}^{6}}{36}}}{L^{2}}\left(1-\frac{x_{3}}{2L}\sqrt{\frac{E}{4G}}\right)$$

(163) $$P=6000\;\mathrm{lb},\;\;\;\;\;\;L=14\;\mathrm{in},\;\;\;\;\;\;\delta_{\max}=0.25\;\mathrm{in}$$

(164) $$E=30\times 10^{6}\;\mathrm{psi},\;\;\;\;\;\;G=12\times 10^{6}\;\mathrm{psi}$$

(165) $$\tau_{\max}=13600\;\mathrm{psi},\;\;\;\;\;\;\sigma =30000\;\mathrm{psi}$$

Variable range

(166) $$0.1\leq x_{1},x_{4}\leq 2,\;\;\;\;\;\;0.1\leq x_{2},x_{3}\leq 10$$

Table 17 delineates the quantitative results of the welden beam. The real part inherits the CGCRA foraging and gathering strategy to emphasize local exploitation, fine-tuning weld and beam sizes to minimize cost while maintaining structural integrity. The imaginary part harnesses CGCRA random migration and perturbation mutation to perpetually explore uncharted territories and achieve concurrent exploration and exploitation of the same individual. The CGCRA enriches and balances the information transmission and collaborative search between GCR individuals through a complex-valued operation and encoding form, which can effectively suppress the random factors’ interference, reduce the outliers’ probability, and enhance stability and repeatability. The CGCRA has strong stability and repeatability to determine the optimum variables and cost.

## 6. Conclusions and Future Research

This paper presents the CGCRA with a dual-encoding redundancy mechanism to tackle benchmark functions and engineering designs; the purpose is to quantitatively assess the convergence efficiency, solution precision, stability, and repeatability of the CGCRA by minimizing the fitness, comprehensively facilitating the engineering quality and system reliability by optimizing the control variables and optimum solutions. The complex-valued encoding strategy is integrated into the GCRA to encounter some drawbacks of languid convergence speed, inadequate calculation precision, susceptibility to the local optimum, noteworthy dimensionality disaster, and a tendency towards imbalance between prospecting and mining. The CGCRA utilizes the information interaction and collaborative search mechanism between GCR individuals of the complex-valued encoding mechanism to achieve the optimal foraging areas and trails, which augments information capacity and population variety, advances investigated flexibility and anti-interference ability, promotes parallel computation and exploration efficiency, and fosters spatial transformation and multi-dimensional data processing, mitigates local optimality and random factors interference, and facilitates consistency and repeatability. In the exploration phase, the GCRs evacuate the numerous refuges within the territory to scavenge and establish trails, which facilitates a comprehensive investigation and restricts pursuit standstill to cultivate the multiple potential optimum solutions. In the exploitation phase, the male GCRs isolate themselves from the population during the breeding period and specialize in foraging within regions of abundant food sources, which reinforces the local search and exploits refined optimization to upgrade the precision and quality of the optimum solutions. The CGCRA exhibits instructive flexibility and adaptability in incorporating CGCRA’s multidimensional data of the resource distribution and characterizing the GCRS trade-off relationships of the optimal foraging areas and trails, accomplishing a dynamic equilibrium and collaborative pursuit. The CGCRA is contrasted with the BKA, EGO, HLOA, IAO, NRBO, SBOA, WSA, EHO, WO, HEOA, PO, and GCRA. The experimental results demonstrate that the CGCRA not only endures remarkable flexibility and adaptability in mitigating search stagnation and recognizing the optimum solution but also exhibits substantial repeatability and anti-interference to furnish accelerated convergence speed and heightened computational precision.

The CGCRA exhibits the following limitations: (1) the CGCRA has high complex-valued encoding complexity and cumbersome decoding operation, which increases storage space and computing resource burden, introduces redundant detection information, reduces execution efficiency and convergence accuracy, and affects the algorithm’s versatility and stability. (2) The CGCRA faces serious potential threats in terms of the parameter sensitivity, parameter interaction, and parameter setting. Based on the encoding particularity, algorithm complexity, and parameter interaction, we usually need a lot of experiments and debugging to determine the appropriate parameters in the absence of effective parameter tuning methods and theoretical guidance. (3) The CGCRA has poor adaptability and consistency in addressing the special structural issues with non-conveys, discontinuous characteristics, non-smooth objective functions, discrete optimization, integer programming, symmetry, periodicity, and strong constraints, which requires additional transformations or special processing, increases computational complexity and implementation difficulty, inhibits the algorithm’s feasibility and reliability, and provides local optimality and poor convergence solution. (4) Based on the different encoding forms and the algorithm’s structure principles, the efficient combination of the CGCRA and other real encoding algorithms requires complex spatial transformation and mapping to achieve information interaction and algorithm fusion, which may cause search conflicts or mutual interference, lose mutual cooperative information, reduce parallel computing efficiency, unbalance exploration and exploitation, enhance parameter sensitivity, increase fluctuation risks, limit scalability and parallelism, and affect search efficiency and convergence accuracy.

Our future research will be directed toward the following areas: (1) efficient detection strategies (e.g., multitransfer strategy, multi-population evolution strategy, regeneration strategy, conflict-based strategy, Cauchy noise escape strategy, adaptive perturbation factor, adaptive growth rate), significant encoding forms (e.g., binary, numerical, polar coordinates, structural, quantum, symbol, Gray, matrix, discrete, interval, sequential, probability encoding), complimentary hybrid approaches (e.g., dream optimization algorithm, arctic puffin optimization, black-winged kite algorithm, blood-sucking leech optimizer, elk herd optimizer, horned lizard defense tactics) will be used to promote the consistency and reliability, accelerate convergence efficiency, and enhance the computational accuracy. (2) The CGCRA will be used to achieve the understory crops’ multi-equipment coordinated operation scheduling and resource allocation, intelligent navigation with high-precision positioning and dynamic obstacle avoidance in complex terrains, multi-source data information fusion and multi-target intelligent decision-making, multi-device collaborative communication and human-machine collaborative efficient operation, fault warning and maintenance decision-making, information perception, and pest control. (3) The CGCRA will be extended to address the dynamic and high-multilevel thresholding in color image segmentation. Meanwhile, the CGCRA will be used to explore deep learning to bolster the training efficiency and generalization performance of neural networks, such as model structure search, hyperparameter adjustment, and deep fusion of complex-valued neural networks.

## Figures and Tables

**Table 1:** Cross-dimensional analysis of four categories.

Comparative Dimension	Swarm Intelligence	Evolutionary Computation	Physics/Chemistry/Mathematics-Inspired Optimizers	Human Behavior-Inspired Approaches
Inspiration source	Individual collaboration and information sharing, foraging, predation, migration, and division	Biological genetics, variation, natural selection, species evolution, survival of the fittest	Physical phenomena, chemical reactions, inherent mathematical properties	Human social behavior, cognitive decision-making patterns, cultural evolution characteristics, brainstorming
Operational logic	Individual self-organizing collaboration, relying on individual historical optimality and population global optimality to guide solution refresh	Realize intergenerational evolution and promote the population towards an optimal direction through selection, crossover, and mutation	Physical energy/force field changes, chemical reaction logic; mathematical rules of neighborhood search, taboo, perturbation	Imitate human trial and error, individual learning, group communication, experience reference to complete iterative updates of solutions
Exploration and exploitation tendency	Overall balance, classic algorithms have excellent balance, new algorithms tend to explore globally	Strong global exploration, moderate local exploitation	Physics/chemistry: strong exploration, weak exploitation, pure mathematics: strong exploitation, weak exploration	Balance exploration and exploitation, flexible dynamic adjustment
Information interaction	Individual high-frequency interaction, global optimal dominance, high interaction intensity	Intergenerational cross fusion, global information sharing, medium to high interaction intensity	Physics/chemistry: particle indirect field interaction, low intensity, pure mathematics: no interaction	Imitative weak interaction without strong global guidance
Parameter sensitivity	Medium parameter scale, most parameters are sensitive, parameter tuning relies on experience	Medium parameter scale, crossover and variation parameters have a significant impact on performance	Physics/chemistry: multiple parameters, high sensitivity, pure mathematics: few parameters	Few parameters, some algorithms have no additional adjustable parameters
Distinctive merits	Good parallelism, strong robustness, compatible with continuous/combinatorial optimization problems	Highly versatile, adaptable to continuous, discrete, and mixed variables, flexible coding, mature theory	Physics/chemistry: diversified ways to escape from local optima, pure mathematics: simple implementation, fast speed, high local accuracy	Intuitive inspiration, simple rules, low entry barriers, strong engineering practicality, few parameters
Insufficient defects	Ubiquitous metaphorical abstraction, high-dimensional premature convergence, limited generality, easy search stagnation, weak theoretical foundation	Slow convergence speed, weak local mining, easy to fall into local optima, insufficient diversity	Physics/chemistry: metaphorically stiff, poorly interpretable, pure mathematics: search delay, not suitable for large-scale high-dimensional applications	Excessive proliferation of metaphors, severe homogenization, weak theoretical foundation, lack of convergence proof, performance degradation in strongly constrained scenarios
Applicable scenarios	Continuous optimization, combinatorial optimization, large-scale high-dimensional multimodal optimization	Suitable for continuous optimization, parameter optimization, feature selection, automatic program generation	Suitable for multi-modal global optimization and combinatorial optimization	Suitable for combining optimization with prior knowledge or memory mechanisms (travel agents, scheduling)

**Table 2:** Chromosomes of the complex natural individual organisms.

*Individual*	$Individual_{1}$	$Individual_{2}$	$Individual_{i}$	…	$Individual_{M}$
Real part $R_{P}$	$R_{P1}$	$R_{P2}$	$R_{Pi}$		$R_{PM}$
Imaginary part $I_{P}$	$I_{P1}$	$I_{P2}$	$I_{Pi}$	…	$I_{PM}$
Chromosome structure	$\left(R_{P1},I_{P1}\right)$	$\left(R_{P2},I_{P2}\right)$	$\left(R_{Pi},I_{Pi}\right)$	…	$\left(R_{PM},I_{PM}\right)$

**Table 3:** Benchmark functions.

Benchmark Functions	Dim	Range	$f_{\min}$
$f_{1}=\sum_{i=1}^{n}x_{i}^{2}$	30	[−100, 100]	0
$f_{2}(x)=\sum_{i=1}^{n}\left|x_{i}\right|+\prod_{i=1}^{n}\left|x_{i}\right|$	30	[−10, 10]	0
$f_{3}(x)=\sum_{i=1}^{n}(\sum_{j=1}^{i}x_{j}{)}^{2}$	30	[−100, 100]	0
$f_{4}(x)=\max_{i}\{|x_{i}|,1\leq i\leq n\}$	30	[−100, 100]	0
$f_{5}(x)=\sum_{i=1}^{n-1}\left[100{\left(x_{i+1}-x_{i}^{2}\right)}^{2}+{\left(x_{i}-1\right)}^{2}\right]$	30	[−30, 30]	0
$f_{6}(x)=\sum_{i=1}^{n}{\left([x_{i}+0.5]\right)}^{2}$	30	[−100, 100]	0
$f_{7}(x)=\sum_{i=1}^{n}ix_{i}^{4}+random[0,1)$	30	[−1.28, 1.28]	0
$f_{8}(x)=\sum_{i=1}^{n}\left[x_{i}^{2}-10\cos(2\pi x_{i})+10\right]$	30	[−5.12, 5.12]	0
$\begin{array}{l} f_{9}(x)=-20\exp\left(-0.2\sqrt{\frac{1}{n}\sum_{i=1}^{n}x_{i}^{2}}-\exp\left(\frac{1}{n}\sum_{i=1}^{n}\cos2\pi x_{i}\right)\right)\\ +20+e \end{array}$	30	[−32, 32]	0
$f_{10}(x)=\frac{1}{4000}\sum_{i=1}^{n}\left(x_{i}^{2}\right)-\prod_{i=1}^{n}\cos\left(\frac{x_{i}}{\sqrt{i}}\right)+1$	30	[−600, 600]	0
$\begin{array}{l} f_{11}(x)=\frac{\pi }{n}\left\{10\sin^{2}(\pi y_{1})+\sum_{i=1}^{n-1}\begin{array}{l} {\left(y-1\right)}^{2}[1+10\sin^{2}(\pi y_{1})]\\ +{\left(y_{n}-1\right)}^{2} \end{array}\right\}\\ +\sum_{i=1}^{n}u(x_{i},10,100,4)\\ y_{i}=1+\frac{x_{i}+1}{4}\\ u(x_{i},a,k,m)=\left\{\begin{array}{l} k{\left(x_{i}-a\right)}^{m},x^{i}>a\\ 0,-a\leq x_{i}\leq a\\ k{\left(-x_{i}-z\right)}^{m},x_{i}<a \end{array}\right. \end{array}$	30	[−50, 50]	0
$\begin{array}{l} f_{12}(x)=0.1\left\{\sin^{2}\left(3\pi x_{1}+\sum_{i=1}^{n}\begin{array}{l} {\left(x_{i}-1\right)}^{2}[1+\sin^{2}(3\pi x_{i}+1)]\\ +{\left(x_{n}-1\right)}^{2}[1+\sin^{2}(2\pi x_{n})] \end{array}\right)\right\}\\ +\sum_{i=1}^{n}u(x_{i},5,100,4) \end{array}$	30	[−50, 50]	0
$f_{13}(x)=(\frac{1}{500}+\sum_{j=1}^{25}\frac{1}{j+\sum_{i=1}^{2}{\left(x_{i}-a_{ij}\right)}^{6}}{)}^{-1}$	2	[−65, 65]	0.998
$f_{14}(x)=\sum_{i=1}^{11}{\left[a_{i}-\frac{x_{1}(b_{i}^{2}+b_{i}x_{2})}{b_{i}^{2}+b_{i}x_{3}+x_{4}}\right]}^{2}$	4	[−5, 5]	0.000307
$f_{15}(x)=4x_{1}^{2}-2.1x_{1}^{4}+\frac{1}{3}x_{1}^{6}+x_{1}x_{2}-4x_{2}^{2}+4x_{2}^{4}$	2	[−5, 5]	−1.0316
$f_{16}(x)=-\frac{1+\cos(12\sqrt{x_{1}^{2}+x_{2}^{2}})}{0.5(x_{1}^{2}+x_{2}^{2})+2}$	2	[−5.12, 5.12]	−1
$\begin{array}{l} f_{17}(x)=[1+{\left(x_{1}+x_{2}+1\right)}^{2}\left(19-14x_{1}+3x_{1}^{2}-14x_{2}+6x_{1}x_{2}+3x_{2}^{2}\right)]\\ \times [30+{\left(2x_{1}-3x_{2}\right)}^{2}(18-32x_{1}+12x_{1}^{2}+48x_{2}-36x_{1}x_{2}+27x_{2}^{2})] \end{array}$	2	[−2, 2]	3
$f_{18}(x)=-\sum_{i=1}^{5}{\left[(x-a_{i}){\left(x-a_{i}\right)}^{T}+c_{i}\right]}^{-1}$	4	[0, 10]	−10.1532
$f_{19}(x)=-\sum_{i=1}^{7}{\left[(x-a_{i}){\left(x-a_{i}\right)}^{T}+c_{i}\right]}^{-1}$	4	[0, 10]	−10.4029
$f_{20}(x)=-\sum_{i=1}^{10}{\left[(x-a_{i}){\left(x-a_{i}\right)}^{T}+c_{i}\right]}^{-1}$	4	[0, 10]	−10.5364
$f_{21}(x)=-\cos(x_{1})\cos(x_{2})\exp(-{\left(x_{1}-\pi \right)}^{2}-{\left(x_{2}-\pi \right)}^{2})$	2	[−2π,2π]	−1
$f_{22}(x)=0.5+\frac{\sin^{2}\left(\sqrt{x_{1}^{2}+x_{2}^{2}}\right)-0.5}{{\left(1+0.001(x_{1}^{2}+x_{2}^{2})\right)}^{2}}$	2	[−100, 100]	−1
$f_{23}(x)=\sum_{i=1}^{n}x_{i}\sin(x_{i})+0.1x_{i}$	10	[−10, 10]	0

**Table 4:** Quantitative results of benchmark functions.

Function	Result	BKA	EGO	HLOA	IAO	NRBO	SBOA	WSA	EHO	WO	HEOA	PO	GCRA	CGCRA
$f_{1}$	Best	1.0 × 10^−218^	0	0	0	0	0	0	5.83 × 10^−22^	0	1.2 × 10^−200^	0	0	0
	Worst	2.8 × 10^−183^	0	0	0	0	0	0	4.01 × 10^−17^	0	5.7 × 10^−158^	0	0	0
	Mean	9.4 × 10^−185^	0	0	0	0	0	0	1.47 × 10^−18^	0	1.9 × 10^−159^	0	0	0
	Std	0	0	0	0	0	0	0	7.31 × 10^−18^	0	1.0 × 10^−158^	0	0	0
$f_{2}$	Best	8.9 × 10^−112^	0	3.2 × 10^−263^	0	0	5.1 × 10^−185^	2.6 × 10^−157^	3.14 × 10^−14^	2.9 × 10^−196^	2.81 × 10^−92^	9.8 × 10^−277^	0	0
	Worst	1.60 × 10^−98^	0	4.4 × 10^−244^	0	1.6 × 10^−305^	4.1 × 10^−164^	6.1 × 10^−152^	3.06 × 10^−10^	4.0 × 10^−167^	3.96 × 10^−79^	2.4 × 10^−266^	0	0
	Mean	5.3 × 10^−100^	0	1.5 × 10^−245^	0	5.8 × 10^−307^	1.4 × 10^−165^	2.5 × 10^−153^	1.16 × 10^−11^	1.4 × 10^−168^	1.52 × 10^−80^	9.6 × 10^−268^	0	0
	Std	2.90 × 10^−99^	0	0	0	0	0	1.1 × 10^−152^	5.57 × 10^−11^	0	7.22 × 10^−80^	0	0	0
$f_{3}$	Best	3.5 × 10^−216^	0	0	0	0	1.4 × 10^−241^	0	0.971331	0	9.7 × 10^−215^	0	0	0
	Worst	6.6 × 10^−176^	0	0	0	0	1.0 × 10^−206^	3.7 × 10^−304^	41.9567	0	6.5 × 10^−207^	0	0	0
	Mean	2.2 × 10^−177^	0	0	0	0	3.5 × 10^−208^	1.2 × 10^−305^	8.686946	0	4.0 × 10^−208^	0	0	0
	Std	0	0	0	0	0	0	0	10.34673	0	0	0	0	0
$f_{4}$	Best	3.7 × 10^−108^	0	2.1 × 10^−269^	0	3.1 × 10^−306^	2.5 × 10^−152^	1.6 × 10^−164^	3.376950	8.8 × 10^−187^	4.34 × 10^−58^	1.1 × 10^−276^	0	0
	Worst	3.90 × 10^−80^	0	9.6 × 10^−246^	0	5.7 × 10^−298^	3.8 × 10^−132^	4.0 × 10^−153^	21.59668	1.3 × 10^−155^	4.64 × 10^−48^	1.2 × 10^−268^	0	0
	Mean	1.30 × 10^−81^	0	3.2 × 10^−247^	0	2.6 × 10^−299^	1.6 × 10^−133^	3.4 × 10^−154^	10.60969	4.2 × 10^−157^	2.30 × 10^−49^	4.9 × 10^−270^	0	0
	Std	7.11 × 10^−81^	0	0	0	0	7.1 × 10^−133^	9.8 × 10^−154^	4.459371	2.3 × 10^−156^	8.94 × 10^−49^	0	0	0
$f_{5}$	Best	24.20335	26.05616	1.72 × 10^−6^	28.67618	26.37176	22.95210	15.95264	3.374669	2.83 × 10^−7^	0.233185	0.002288	2.75 × 10^−10^	0
	Worst	28.91707	28.74295	28.70629	28.80147	28.80720	23.85777	20.44314	85.53233	0.039644	3.088819	25.58633	2.16 × 10^−5^	5.97 × 10^−30^
	Mean	25.97674	27.07346	22.94440	28.72847	27.55347	23.38635	18.37509	45.34290	0.006135	0.820575	23.90461	4.33 × 10^−6^	5.77 × 10^−31^
	Std	1.015639	0.623372	11.66838	0.023729	0.836825	0.253120	1.114409	29.87031	0.009105	0.610651	4.540633	5.28 × 10^−6^	1.54 × 10^−30^
$f_{6}$	Best	3.12 × 10^−5^	3.128740	2.28 × 10^−6^	0.166554	1.492294	1.14 × 10^−15^	0	4.63 × 10^−22^	1.84 × 10^−8^	0.000351	3.46 × 10^−11^	8.46 × 10^−11^	3.30 × 10^−27^
	Worst	3.996742	5.048849	0.000384	2.628649	3.291359	1.69 × 10^−13^	0	4.20 × 10^−19^	0.000227	1.020627	1.37 × 10^−8^	1.12 × 10^−6^	1.77 × 10^−21^
	Mean	0.331191	4.137628	9.60 × 10^−5^	0.749069	2.270967	2.66 × 10^−14^	0	5.96 × 10^−20^	4.38 × 10^−5^	0.125299	2.17 × 10^−9^	7.51 × 10^−8^	1.07 × 10^−22^
	Std	0.871799	0.432878	9.28 × 10^−5^	0.530287	0.412345	3.56 × 10^−14^	0	9.02 × 10^−20^	5.61 × 10^−5^	0.204138	3.20 × 10^−9^	2.06 × 10^−7^	3.51 × 10^−22^
$f_{7}$	Best	1.13 × 10^−5^	3.01 × 10^−7^	9.79 × 10^−6^	1.17 × 10^−6^	7.41 × 10^−6^	2.79 × 10^−5^	7.15 × 10^−6^	0.015880	9.22 × 10^−6^	3.85 × 10^−6^	1.62 × 10^−6^	3.08 × 10^−6^	2.52 × 10^−8^
	Worst	0.000320	4.22 × 10^−5^	0.000433	0.000108	0.000355	0.000404	0.000114	0.109753	0.000756	0.000416	0.000179	0.000134	1.32 × 10^−5^
	Mean	9.57 × 10^−5^	1.41 × 10^−5^	0.000116	2.01 × 10^−5^	9.64 × 10^−5^	0.000152	4.68 × 10^−5^	0.052797	0.000123	7.35 × 10^−5^	5.68 × 10^−5^	4.11 × 10^−5^	2.87 × 10^−6^
	Std	6.39 × 10^−5^	1.35 × 10^−5^	0.000114	2.09 × 10^−5^	8.05 × 10^−5^	9.22 × 10^−5^	2.95 × 10^−5^	0.026017	0.000143	8.82 × 10^−5^	5.13 × 10^−5^	2.87 × 10^−5^	3.89 × 10^−6^
$f_{8}$	Best	0	0	0	0	0	0	0	14.92438	0	29.43536	0	0	0
	Worst	0	0	0	0	0	0	0	45.76807	0	52.11550	0	0	0
	Mean	0	0	0	0	0	0	0	28.38948	0	31.08455	0	0	0
	Std	0	0	0	0	0	0	0	7.024402	0	4.451099	0	0	0
$f_{9}$	Best	4.44 × 10^−16^	4.44 × 10^−16^	4.44 × 10^−16^	4.44 × 10^−16^	4.44 × 10^−16^	4.44 × 10^−16^	4.44 × 10^−16^	3.39 × 10^−11^	4.44 × 10^−16^	4.44 × 10^−16^	4.44 × 10^−16^	4.44 × 10^−16^	4.44 × 10^−16^
	Worst	4.44 × 10^−16^	4.44 × 10^−16^	4.44 × 10^−16^	4.44 × 10^−16^	4.44 × 10^−16^	4.44 × 10^−16^	4.44 × 10^−16^	5.055769	4.44 × 10^−16^	4.44 × 10^−16^	4.44 × 10^−16^	4.44 × 10^−16^	4.44 × 10^−16^
	Mean	4.44 × 10^−16^	4.44 × 10^−16^	4.44 × 10^−16^	4.44 × 10^−16^	4.44 × 10^−16^	4.44 × 10^−16^	4.44 × 10^−16^	2.130817	4.44 × 10^−16^	4.44 × 10^−16^	4.44 × 10^−16^	4.44 × 10^−16^	4.44 × 10^−16^
	Std	0	0	0	0	0	0	0	0.994608	0	0	0	0	0
$f_{10}$	Best	0	0	0	0	0	0	0	0	0	0	0	0	0
	Worst	0	0	0	0	0	0	0	0.319056	0	0.333289	0	0	0
	Mean	0	0	0	0	0	0	0	0.022654	0	0.033246	0	0	0
	Std	0	0	0	0	0	0	0	0.059112	0	0.065364	0	0	0
$f_{11}$	Best	2.80 × 10^−6^	0.242068	8.17 × 10^−7^	0.008252	0.102783	2.01 × 10^−17^	1.34 × 10^−30^	9.35 × 10^−20^	2.75 × 10^−9^	0.000981	1.24 × 10^−11^	2.88 × 10^−13^	1.57 × 10^−32^
	Worst	0.803968	0.540176	0.103695	0.223576	0.368533	2.81 × 10^−15^	0.207317	1.255514	7.37 × 10^−6^	0.734796	1.51 × 10^−9^	1.11 × 10^−8^	1.57 × 10^−32^
	Mean	0.086193	0.395390	0.006917	0.078206	0.191518	3.68 × 10^−16^	0.024189	0.280662	5.04 × 10^−7^	0.159756	1.75 × 10^−10^	1.54 × 10^−9^	1.57 × 10^−32^
	Std	0.195793	0.081230	0.026304	0.048780	0.061997	5.29 × 10^−16^	0.052247	0.379074	1.35 × 10^−6^	0.224742	2.74 × 10^−10^	2.36 × 10^−9^	5.57 × 10^−48^
$f_{12}$	Best	0.111943	0.117460	3.93 × 10^−6^	0.124931	1.403198	5.02 × 10^−16^	7.73 × 10^−31^	1.92 × 10^−22^	3.02 × 10^−8^	5.83 × 10^−5^	2.81 × 10^−11^	2.27 × 10^−11^	1.35 × 10^−32^
	Worst	2.995549	1.026511	0.463738	2.980248	2.979620	0.196254	0.010987	3.597465	4.44 × 10^−5^	0.004473	0.010987	1.70 × 10^−7^	1.35 × 10^−32^
	Mean	1.315111	0.539558	0.022082	2.444998	1.994001	0.032608	0.001099	0.402937	5.93 × 10^−6^	0.001439	0.000366	3.35 × 10^−8^	1.35 × 10^−32^
	Std	0.741137	0.225103	0.085605	1.000037	0.419754	0.053517	0.003353	1.021875	9.18 × 10^−6^	0.001230	0.002006	4.60 × 10^−8^	5.57 × 10^−48^
$f_{13}$	Best	0.998004	0.998004	0.998004	0.998004	0.998004	0.998004	0.998004	0.998004	0.998004	0.998004	0.998004	0.998004	0.998004
	Worst	2.982105	1.038265	12.67051	0.998004	2.982105	0.998004	1.992031	5.928845	0.998004	12.67051	0.998004	0.998004	0.998004
	Mean	1.064141	0.999779	4.725066	0.998004	1.593234	0.998004	1.031138	2.184934	0.998004	7.534543	0.998004	0.998004	0.998004
	Std	0.362246	0.007372	3.861381	0	0.924773	0	0.181484	1.668560	3.02 × 10^−16^	5.268548	0	2.20 × 10^−12^	1.24 × 10^−16^
$f_{14}$	Best	0.000307	0.000308	0.000307	0.000307	0.000307	0.000307	0.000307	0.000307	0.000308	0.000308	0.000307	0.000675	0.000307
	Worst	0.020363	0.000367	0.095598	0.000307	0.020363	0.020363	0.001223	0.001643	0.000429	0.000325	0.001223	0.001674	0.000307
	Mean	0.001140	0.000316	0.006857	0.000307	0.003139	0.002374	0.000521	0.000742	0.000315	0.000314	0.000369	0.001619	0.000307
	Std	0.003657	1.17 × 10^−5^	0.018388	1.27 × 10^−19^	0.006880	0.006103	0.000394	0.000383	2.23 × 10^−5^	4.77 × 10^−6^	0.000232	0.000215	1.15 × 10^−9^
$f_{15}$	Best	−1.03163	−1.03161	−1.03163	−1.03163	−1.03163	−1.03163	−1.03163	−1.03163	−1.03163	−1.03163	−1.03163	−1.03070	−1.03163
	Worst	−1.03163	−1.03034	−1.03163	−1.03163	−1.03163	−1.03163	−1.03163	−1.03163	−1.02868	−1.03138	−1.03163	−0.76772	−1.03163
	Mean	−1.03163	−1.03131	−1.03163	−1.03163	−1.03163	−1.03163	−1.03163	−1.03163	−1.03150	−1.03157	−1.03163	−0.97326	−1.03163
	Std	6.58 × 10^−16^	0.000276	5.13 × 10^−16^	6.78 × 10^−16^	6.05 × 10^−16^	6.78 × 10^−16^	6.32 × 10^−16^	6.78 × 10^−16^	0.000548	6.71 × 10^−5^	6.65 × 10^−16^	0.061576	6.58 × 10^−16^
$f_{16}$	Best	−1	−1	−1	−1	−1	−1	−1	−1	−1	−1	−1	−1	−1
	Worst	−1	−1	−1	−1	−1	−1	−1	−0.93625	−1	−1	−1	−1	−1
	Mean	−1	−1	−1	−1	−1	−1	−1	−0.98512	−1	−1	−1	−1	−1
	Std	0	0	0	0	0	0	0	0.027426	0	0	0	1.66 × 10^−11^	0
$f_{17}$	Best	3	3	3	3	3	3	3	3	3	3.000008	3	3.02067	3
	Worst	3	3.000452	3	3	3	3	3	3	3	4.07082	3	32.68452	3
	Mean	3	3.000070	3	3	3	3	3	3	3	3.041238	3	15.85923	3
	Std	1.49 × 10^−15^	0.000113	2.86 × 10^−14^	1.88 × 10^−15^	4.26 × 10^−15^	5.34 × 10^−16^	1.57 × 10^−15^	2.91 × 10^−15^	1.77 × 10^−14^	0.195209	2.20 × 10^−15^	11.36096	1.27 × 10^−15^
$f_{18}$	Best	−10.1532	−9.66200	−10.1532	−10.1532	−10.1532	−10.1532	−10.1532	−10.1532	−10.1532	−10.1522	−10.1532	−10.1532	−10.1532
	Worst	−10.1532	−5.02191	−2.63047	−5.05520	−2.63047	−10.1532	−5.05520	−2.68286	−10.1532	−5.98821	−10.1532	−10.1512	−10.1527
	Mean	−10.1532	−5.64063	−9.40027	−6.24473	−9.57780	−10.1532	−9.30353	−9.06934	−10.1532	−9.12428	−10.1532	−10.1529	−10.1531
	Std	5.67 × 10^−15^	1.550303	2.295176	2.193074	1.702071	6.68 × 10^−15^	1.932393	2.513613	6.02 × 10^−12^	1.153430	6.62 × 10^−15^	0.000462	0.000149
$f_{19}$	Best	−10.4029	−5.08668	−10.4029	−10.4029	−10.4029	−10.4029	−10.4029	−10.4029	−10.4029	−10.4023	−10.4029	−10.4028	−10.4028
	Worst	−10.4029	−5.03131	−2.76590	−5.08767	−2.76590	−5.08767	−5.08767	−2.75193	−10.4029	−6.83463	−10.4029	−10.3993	−10.4020
	Mean	−10.4029	−5.07580	−9.16103	−8.27683	−10.0572	−10.2258	−9.16271	−9.92529	−10.4029	−9.64218	−10.4029	−10.4024	−10.4027
	Std	1.23 × 10^−15^	0.011622	2.826183	2.648454	1.405289	0.970431	2.286539	1.822252	3.35 × 10^−12^	0.84445	8.08 × 10^−16^	0.000679	0.000196
$f_{20}$	Best	−10.5364	−10.1955	−10.5364	−10.5364	−10.5364	−10.5364	−10.5364	−10.5364	−10.5364	−10.5339	−10.5364	−10.5363	−10.5363
	Worst	−10.5364	−5.06838	−2.42173	−5.12848	−8.12759	−10.5364	−5.12848	−2.87114	−10.5364	−7.37302	−10.5364	−10.5310	−10.5351
	Mean	−10.5364	−5.44653	−9.50118	−6.39033	−10.3891	−10.5364	−9.63509	−9.57866	−10.5364	−9.81742	−10.5364	−10.5357	−10.5361
	Std	2.88 × 10^−15^	1.265408	2.692296	2.326399	0.561864	2.03 × 10^−15^	2.04987	2.489983	1.63 × 10^−11^	0.939153	2.36 × 10^−15^	0.001133	0.000311
$f_{21}$	Best	−1	−1	−1	−1	−1	−1	−1	−1	−1	−1	−1	−1	−1
	Worst	−1	−0.99956	−1	−1	−1	−1	−1	−1	−1	−0.99994	−1	−1	−1
	Mean	−1	−0.99989	−1	−1	−1	−1	−1	−1	−1	−0.99999	−1	−1	−1
	Std	0	0.000110	0	0	0	0	0	0	0	1.31 × 10^−5^	0	2.33 × 10^−7^	7.75 × 10^−7^
$f_{22}$	Best	−1	−1	−1	−1	−1	−1	−1	−1	−1	−1	−1	−1	−1
	Worst	−1	−1	−1	−1	−1	−1	−1	−0.99028	−1	−0.99028	−0.99028	−1	−1
	Mean	−1	−1	−1	−1	−1	−1	−1	−0.99158	−1	−0.99644	−0.99968	−1	−1
	Std	0	0	0	0	0	0	0	0.003359	0	0.004762	0.001774	6.82 × 10^−10^	0
$f_{23}$	Best	1.8 × 10^−111^	0	2.3 × 10^−282^	0	0	4.7 × 10^−232^	2.2 × 10^−180^	3.4 × 10^−131^	3.8 × 10^−205^	5.66 × 10^−96^	5.3 × 10^−273^	4.88 × 10^−13^	0
	Worst	3.50 × 10^−79^	0	2.3 × 10^−239^	0	0	1.16 × 10^−31^	1.93 × 10^−5^	2.05 × 10^−15^	1.8 × 10^−171^	0.313260	3.7 × 10^−264^	1.48 × 10^−5^	0
	Mean	1.17 × 10^−80^	0	7.7 × 10^−241^	0	0	4.30 × 10^−33^	2.47 × 10^−6^	3.53 × 10^−16^	6.2 × 10^−173^	0.025821	3.1 × 10^−265^	1.12 × 10^−6^	0
	Std	6.39 × 10^−80^	0	0	0	0	2.12 × 10^−32^	5.50 × 10^−6^	5.27 × 10^−16^	0	0.072874	0	3.44 × 10^−6^	0

**Table 5:** Quantitative results of Wilcoxon rank-sum test on the benchmark functions.

Function	CGCRA vs. BKA	CGCRA vs. EGO	CGCRA vs. HLOA	CGCRA vs. IAO	CGCRA vs. NRBO	CGCRA vs. SBOA	CGCRA vs. WSA	CGCRA vs. EHO	CGCRA vs. WO	CGCRA vs. HEOA	CGCRA vs. PO	CGCRA vs. GCRA
$f_{1}$	1.21 × 10^−12^	N/A	N/A	N/A	N/A	N/A	N/A	1.21 × 10^−12^	N/A	1.21 × 10^−12^	N/A	N/A
$f_{2}$	1.21 × 10^−12^	N/A	1.21 × 10^−12^	N/A	2.15 × 10^−2^	1.21 × 10^−12^	1.21 × 10^−12^	1.21 × 10^−12^	1.21 × 10^−12^	1.21 × 10^−12^	1.21 × 10^−12^	N/A
$f_{3}$	1.21 × 10^−12^	N/A	N/A	N/A	N/A	1.21 × 10^−12^	1.60 × 10^−2^	1.21 × 10^−12^	N/A	1.21 × 10^−12^	N/A	N/A
$f_{4}$	1.21 × 10^−12^	N/A	1.21 × 10^−12^	N/A	1.21 × 10^−12^	1.21 × 10^−12^	1.21 × 10^−12^	1.21 × 10^−12^	1.21 × 10^−12^	1.21 × 10^−12^	1.21 × 10^−12^	N/A
$f_{5}$	6.46 × 10^−12^	6.46 × 10^−12^	6.46 × 10^−12^	6.46 × 10^−12^	6.46 × 10^−12^	6.46 × 10^−12^	6.46 × 10^−12^	6.46 × 10^−12^	6.46 × 10^−12^	6.46 × 10^−12^	6.46 × 10^−12^	6.46 × 10^−12^
$f_{6}$	3.02 × 10^−11^	3.02 × 10^−11^	3.02 × 10^−11^	3.02 × 10^−11^	3.02 × 10^−11^	3.02 × 10^−11^	1.21 × 10^−12^	6.07 × 10^−11^	3.02 × 10^−11^	3.02 × 10^−11^	3.02 × 10^−11^	3.02 × 10^−11^
$f_{7}$	3.69 × 10^−11^	1.53 × 10^−5^	4.08 × 10^−11^	6.01 × 10^−8^	8.15 × 10^−11^	3.02 × 10^−11^	1.33 × 10^−10^	3.02 × 10^−11^	6.70 × 10^−11^	1.46 × 10^−10^	6.12 × 10^−10^	1.61 × 10^−10^
$f_{8}$	N/A	N/A	N/A	N/A	N/A	N/A	N/A	1.21 × 10^−12^	N/A	1.21 × 10^−12^	N/A	N/A
$f_{9}$	2.88 × 10^−6^	3.02 × 10^−11^	3.02 × 10^−11^	2.83 × 10^−8^	3.02 × 10^−11^	3.99 × 10^−4^	2.02 × 10^−8^	3.02 × 10^−11^	1.62 × 10^−2^	3.02 × 10^−11^	1.91 × 10^−2^	2.26 × 10^−3^
$f_{10}$	N/A	N/A	N/A	N/A	N/A	N/A	N/A	3.45 × 10^−7^	N/A	1.27 × 10^−5^	N/A	N/A
$f_{11}$	1.21 × 10^−12^	1.21 × 10^−12^	1.21 × 10^−12^	1.21 × 10^−12^	1.21 × 10^−12^	1.21 × 10^−12^	1.21 × 10^−12^	1.21 × 10^−12^	1.21 × 10^−12^	1.21 × 10^−12^	1.21 × 10^−12^	1.21 × 10^−12^
$f_{12}$	1.21 × 10^−12^	1.21 × 10^−12^	1.21 × 10^−12^	1.21 × 10^−12^	1.21 × 10^−12^	1.21 × 10^−12^	1.21 × 10^−12^	1.21 × 10^−12^	1.21 × 10^−12^	1.21 × 10^−12^	1.21 × 10^−12^	1.21 × 10^−12^
$f_{13}$	6.65 × 10^−1^	3.16 × 10^−12^	1.16 × 10^−10^	8.14 × 10^−2^	5.47 × 10^−5^	8.14 × 10^−2^	1.86 × 10^−2^	5.59 × 10^−4^	3.55 × 10^−9^	3.16 × 10^−12^	8.14 × 10^−2^	3.15 × 10^−12^
$f_{14}$	1.50 × 10^−2^	3.02 × 10^−11^	4.20 × 10^−2^	2.68 × 10^−11^	7.72 × 10^−2^	5.56 × 10^−10^	5.19 × 10^−3^	7.01 × 10^−2^	3.02 × 10^−11^	3.02 × 10^−11^	9.34 × 10^−9^	3.02 × 10^−11^
$f_{15}$	N/A	3.15 × 10^−12^	2.49 × 10^−7^	8.14 × 10^−2^	1.58 × 10^−2^	8.14 × 10^−2^	1.73 × 10^−2^	8.14 × 10^−2^	7.72 × 10^−11^	3.15 × 10^−12^	6.54 × 10^−1^	3.15 × 10^−12^
$f_{16}$	N/A	N/A	N/A	N/A	N/A	N/A	N/A	5.46 × 10^−3^	N/A	N/A	N/A	5.37 × 10^−6^
$f_{17}$	7.31 × 10^−1^	1.58 × 10^−11^	1.15 × 10^−10^	2.61 × 10^−5^	3.81 × 10^−2^	5.06 × 10^−3^	8.06 × 10^−2^	5.54 × 10^−4^	3.71 × 10^−11^	1.58 × 10^−11^	5.02 × 10^−3^	1.58 × 10^−11^
$f_{18}$	7.76 × 10^−12^	3.02 × 10^−11^	6.76 × 10^−5^	3.05 × 10^−4^	9.76 × 10^−3^	1.14 × 10^−11^	7.68 × 10^−6^	4.28 × 10^−6^	3.02 × 10^−11^	3.02 × 10^−11^	1.25 × 10^−11^	5.36 × 10^−3^
$f_{19}$	1.33 × 10^−11^	3.02 × 10^−11^	7.48 × 10^−2^	1.83 × 10^−2^	5.71 × 10^−5^	3.15 × 10^−10^	3.66 × 10^−4^	4.23 × 10^−9^	3.02 × 10^−11^	3.69 × 10^−11^	6.32 × 10^−12^	1.44 × 10^−2^
$f_{20}$	2.07 × 10^−11^	3.02 × 10^−11^	3.40 × 10^−2^	3.43 × 10^−4^	9.10 × 10^−6^	4.10 × 10^−12^	8.04 × 10^−6^	3.72 × 10^−7^	3.02 × 10^−11^	3.02 × 10^−11^	7.57 × 10^−12^	1.98 × 10^−2^
$f_{21}$	1.21 × 10^−12^	4.50 × 10^−11^	1.21 × 10^−12^	1.21 × 10^−12^	1.21 × 10^−12^	1.21 × 10^−12^	1.21 × 10^−12^	1.21 × 10^−12^	1.21 × 10^−12^	5.09 × 10^−6^	1.21 × 10^−12^	3.71 × 10^−2^
$f_{22}$	N/A	N/A	N/A	N/A	N/A	N/A	N/A	1.97 × 10^−11^	N/A	1.35 × 10^−4^	3.33 × 10^−2^	1.70 × 10^−8^
$f_{23}$	1.21 × 10^−12^	N/A	1.21 × 10^−12^	N/A	N/A	1.21 × 10^−12^	1.21 × 10^−12^	1.18 × 10^−12^	1.21 × 10^−12^	1.21 × 10^−12^	1.21 × 10^−12^	1.21 × 10^−12^

**Table 6:** Quantitative results of the car side impact.

Algorithm	Optimum Variables						Optimum Weight
	$x_{1}$	$x_{2}$	$x_{3}$	$x_{4}$	$x_{5}$	$x_{6}$	
	$x_{7}$	$x_{8}$	$x_{9}$	$x_{10}$	$x_{11}$		
COA [38]	0.5	1.2791	0.5	1.2739	1.2828	0.5	
	0.5	0.2954	0.192	3.557	19.0792		25.2083
HLOA [38]	0.5	1.0669	0.8016	1.0704	0.504	1.4873	
	0.5	0.192	0.192	−29.9786	3.2119		23.6956
AROA [38]	0.5	1.5	0.5	1.2928	0.5	0.5	
	0.5	0.192	0.3195	8.8265	23.0874		25.3642
ETO [40]	0.50282	1.2414	0.51604	1.2201	0.60334	1.3878	
	0.5	0.74832	0.06747	2.2526	−7.2818		23.2574
SCHO [40]	0.5	1.10286	0.87088	0.88643	0.52609	1.49992	
	0.5	0.03508	0.19439	−30	−0.5913		23.7209
GJO [40]	0.5	1.20309	0.50327	1.28778	0.51053	1.5	
	0.5	0.00000	9.5 × 10^−5^	−22.115	−0.0536		23.4052
SCSO [34]	0.502366774	1.23533939	0.5	1.223008761	0.515267967	1.39187245	
	0.50003369	0.340647775	0.211950171	1.374158706	−7.77399175		23.35787723
SOA [34]	0.500139239	1.254868587	0.5	1.205871077	0.739233716	0.772309974	
	0.5	0.316999014	0.30308334	0.749660043	2.039711514		23.8070425
SFOA [31]	0.5	1.234	0.5	1.187	0.875	0.892	
	0.4	0.345	0.192	1.5	0.572		23.5616
BBO [31]	0.663	1.157	0.5	1.211	0.875	0.882	
	0.4	0.345	0.232	0.726	0.913		23.9386
CGCRA	0.5	1.1165	0.5	1.30214	0.5	1.5	
	0.5	0.345	0.192	−19.57243	0.02436		22.84351

**Table 7:** Quantitative results of the multiple-disc clutch brake.

Algorithm	Optimum Variables					Optimum Weight
	$r_{i}$	$r_{0}$	t	F	Z	
APSO [41]	76	96	1	840	3	0.337181
IAPSO [41]	70	90	1	900	3	0.31365661
GOA [42]	71	92	1	835	3	0.3355146
GSA [43]	72	92	2	815	3	0.3175771
AEO [43]	70	90	1	810	3	0.3136566
AHA [32]	70	90	1	840	3	0.3136566
HBO [44]	70	90	1	1000	3	0.3136566
MRFO [45]	70	90	1	835	3	0.3136566
GA [45]	72	92	1	918	3	0.321498
DE [45]	71	92	1	835	3	0.3355146
CGCRA	70	90	1	600	2	0.23525

**Table 8:** Quantitative results of the rolling element bearing.

Algorithm	Optimum Variables					Optimum Cost
	$D_{m}$	$D_{b}$	Z	$f_{i}$	$f_{o}$	
	$K_{D\min}$	$K_{D\max}$	ε	e	ζ	
HHO [46]	125	21	11.09207	0.515	0.515	
	0.4	0.6	0.3	0.050474	0.6	83,011.88
RSA [46]	125.1722	21.29734	10.88521	0.515253	0.517764	
	0.41245	0.632338	0.301911	0.024395	0.6024	83,486.64
RSO [47]	125	21.41769	10.94027	0.515	0.515	
	0.4	0.7	0.3	0.02	0.6	85,069.021
RUN [48]	125.2142	21.59796	11.4024	0.515	0.515	
	0.40059	0.61467	0.3053	0.02	0.63665	83,680.47
MGA [33]	125.718	21.8745119	10.7770658	0.51500082	0.51500299	
	0.405908353	0.65558802	0.30000415	0.07754492	0.6	83,912.87983
CGO [33]	125	21.875	10.777009	0.515	0.515	
	0.4	0.64620052	0.3	0.050152445	0.6	83,918.49253
EVO [33]	125.7190556	21.4255902	10.6955328	0.515	0.515	
	0.463182936	0.6999265	0.3	0.063431519	0.604213108	81,859.7415974
SELO [49]	126.3521	21.0299	11	0.515	0.515	
	0.4	0.6011	0.3	0.1	0.6004	83,805.29
LFD [49]	126.3999	21	11	0.515	0.5251	
	0.5	0.6	0.3	0.1	0.6	83,670.78
SETO [49]	125.7227	21.4233	11	0.515	0.515	
	0.4	0.7	0.3	0.1	0.6	85,539.19
CGCRA	125.7523	21	11	0.515	0.515	
	0.4513	0.6875	0.3	0.0768	0.6927	85,547.6324

**Table 9:** Quantitative results of the gear train.

Algorithm	Optimum Variables				Optimum Cost
	$n_{A}$	$n_{B}$	$n_{C}$	$n_{D}$	
RSO [37]	39.3326	12	12	25.4127	4.5960 × 10^−8^
ChOA [37]	52.9669	19.2143	16.2824	40.9387	2.2210 × 10^−17^
SOA [37]	58.2626	12	42.0304	60	6.2215 × 10^−16^
STOA [37]	60	24.6745	20.9436	59.696	7.0004 × 10^−15^
TSA [37]	54.0750	18.1623	20.5854	47.9214	5.8199 × 10^−16^
DOA [38]	42.7327	16.2019	19.0945	48.7291	2.7009 × 10^−12^
MSA [38]	33.6104	12.8482	19.9977	52.698	2.3078 × 10^−11^
HLOA [38]	23.4796	12.4617	12.5	47.2468	9.9216 × 10^−10^
MSROA [34]	49.7	19.9	16.5	43.4	2.70 × 10^−12^
SCSO [34]	47.5	26.9	12	46.6	9.92 × 10^−10^
CGCRA	51	22	17	53	3.4762 × 10^−18^

**Table 10:** Quantitative results of three-bar truss.

Algorithm	Optimum Variables		Optimum Weight
	$A_{1}$	$A_{2}$	
BKA [1]	0.788675	0.408248	263.895843
TTAO [35]	0.788688	0.408213	263.8958431
SCHO [50]	0.7886642	0.40827926	263.8958476
APO [39]	0.7887	0.4082	263.89584338
BSLO [51]	0.78867930	0.40823651	263.8958434
FOX [51]	0.78870269	0.4081704	263.8958523
ARSCA [37]	0.7887	0.4081	263.8958
CPO [37]	0.7885	0.4088	263.8959
SBOA [6]	0.789	0.409	264
SFOA [31]	0.78868	0.40825	263.89584
CGCRA	0.78651	0.41362	263.8556

**Table 11:** Quantitative results of the tubular column.

Algorithm	Optimum Variables		Optimum Cost
	d	t	
KOA [36]	5.4512	0.2920	26.499497
FLA [36]	5.4801	0.2905	26.563266
COA [36]	5.4511	0.2920	26.501823
GTO [36]	5.4512	0.2920	26.499497
RUN [36]	5.4512	0.2920	26.499497
SMA [36]	5.4512	0.2920	26.499538
DO [36]	5.4512	0.2920	26.499497
POA [36]	5.4512	0.2920	26.499497
GSA [35]	5.451163397	0.291965509	26.531364472
TTAO [35]	5.452181	0.291626	26.51816147
CGCRA	5.45128	0.29197	24.61613

**Table 12:** Quantitative results of the piston lever.

Algorithm	Optimum Variables				Optimum Weight
	H	B	X	D	
GTO [52]	0.05	2.052859	119.6392	4.089713	8.41270
MFO [52]	0.05	2.041514	120	4.083365	8.412698
WOA [52]	0.051874	2.045915	119.9579	4.085849	8.449975
AOA [53]	0.05	0.125073578	120	4.116042166	7.738
CGO [53]	N/A	N/A	N/A	N/A	8.41281381
MGA [53]	N/A	N/A	N/A	N/A	8.41340665
TTAO [35]	0.05	2.041514	4.083027	120	8.412698323
MVO [2]	0.05	2.046900355	4.095582502	119.92924	8.57509432
ALO [2]	0.05	2.051360067	4.102693186	118.821159	8.53445096
CS-EO [2]	0.05	2.041514	4.083027	120	8.412698
CGCRA	0.05	0.13721	120	4.12642	4.69621

**Table 13:** Quantitative results of the cantilever beam.

Algorithm	Optimum Variables					Optimum Weight
	$x_{1}$	$x_{2}$	$x_{3}$	$x_{4}$	$x_{5}$	
COA [54]	6.01725731	5.30715098	4.49125555	3.508156789	2.149913022	1.33996
APO [39]	6.0160	5.3092	4.4943	3.5015	2.1527	1.33995636
ASO [37]	6.0378	5.3076	4.4870	3.4991	2.1425	1.34
ChOA [37]	5.9364	5.2961	4.4700	3.4297	2.1106	1.3424
GJO [37]	6.0054	5.3041	4.5090	3.4991	2.1562	1.34
RSO [37]	7.2404	4.6475	3.9739	9.1907	1.8512	1.6788
STOA [37]	6.0236	5.3252	4.5124	3.5176	2.0994	1.3402
TSA [37]	5.9426	5.3743	4.4655	3.5156	2.1830	1.3404
CPO [37]	6.0233	5.3196	4.4780	3.5097	2.1436	1.34
HEOA [10]	6.12	5.28	4.46	3.51	2.12	1.34
CGCRA	5.9637	4.8871	4.4753	3.4764	2.1469	1.3068

**Table 14:** Quantitative results of the speed reducer.

Algorithm	Optimum Variables							Optimum Weight
	b	m	z	$l_{1}$	$l_{2}$	$d_{1}$	$d_{2}$	
GTO [39]	3.5	0.7	17	7.3	7.7153	3.3502	5.2867	2994.47106615
APO [39]	3.5	0.7	17	7.3	7.7153	3.3502	5.2867	2994.47106615
BSLO [51]	3.5	0.7	17	7.3	7.71532	3.35021	5.28665	2994.4711
FOX [51]	3.50025	0.7	17	7.30002	7.71587	3.35023	5.28666	2994.525
DOA [38]	3.5012	0.7001	17.0029	7.3461	7.7752	3.3533	5.2890	2999.7
DCS [38]	3.5212	0.7005	17.0096	7.3641	7.8017	3.4337	5.2916	3034.3
HLOA [38]	3.5001	0.7	17.0015	7.3	7.7693	3.3555	5.2867	2999.9
AROA [38]	3.6	0.7	17	7.7798	7.9876	3.3681	5.5	3189.8
EHO [8]	3.5	0.7	17	7.3	7.71532	3.350215	5.286654	2994.471066
ROA [34]	3.4896748	0.7	17	7.822051	7.828591	3.347885	5.278705	2995.685341
CGCRA	3.5	0.7	17	7.3	7.71623	3.35132	5.28664	2994.4234

**Table 15:** Quantitative results of the pressure vessel.

Algorithm	Optimum Variables				Optimum Cost
	$T_{s}$	$T_{h}$	R	L	
BSLO [51]	0.778169	0.3846492	40.31962	200	5885.3328
FOX [51]	0.780559	0.3860622	40.43913	198.5199	5894.5033
EHO [8]	12.450698	6.154387	40.319619	200	5885.332774
ETO [40]	0.7865062	0.3917354	40.54479	199.1287	5984.8509
FLO [55]	0.7780271	0.3845792	40.312284	200	5882.8955
GRO [56]	0.7787153	0.384967	40.347943	199.6061	5886.4068
PKO [57]	0.7781686414	0.3846491626	40.31961872	200	5885.332774
ROA [34]	0.708996562	2.867704095	40.32129317	200	13,048.04256
MSROA [34]	0.773374321	0.374874166	41.83662957	180.1871401	5807.849903
SBOA [6]	0.785	0.388	40.7	195	5890
CGCRA	0.776832	0.373267	39.985692	199.996241	5798.2139

**Table 16:** Quantitative results of the tension/compression spring.

Algorithm	Optimum Variables			Optimum Weight
	d	D	N	
APO [39]	0.0517	0.3567	11.2890	0.01266523
BSLO [51]	0.051669	0.356226	11.31788	0.0126652
FOX [51]	0.051983	0.363808	10.88657	0.0126686
EGO [2]	0.05	0.3157863675	14.29092052	0.0126611
EHO [8]	0.051746	0.358097	11.208557	0.012665
FLO [55]	0.0516891	0.3567177	11.288966	0.0126652
LFD [56]	0.0517	0.3575	11.2442	0.0127
BBOA [56]	0.051344	0.334881	12.6223	0.012667
GRO [56]	0.0517082206	0.35717883	11.2619852	0.012665
SBOA [6]	0.0517	0.357	11.3	0.0127
CGCRA	0.0507	0.3548	11.3736	0.01257

**Table 17:** Quantitative results of the welden beam.

Algorithm	Optimum Variables				Optimum Cost
	h	l	t	b	
BSLO [51]	0.20573	3.47049	9.03662	0.20573	1.72485
FOX [51]	0.20696	3.4519	9.01778	0.20711	1.73142
HOA [8]	0.261132	3.185937	7.846510	0.286110	2.096170
ETO [40]	0.18213	2.4358	9.5821	0.18321	1.4774
FLO [55]	0.2057296	3.4704887	9.0366239	0.2057296	1.7248523
IAO [4]	0.19883	3.33740	9.19200	0.19883	1.67020
PKO [57]	0.2057296398	3.470488666	9.03662391	0.2057296398	1.724852309
MadDE [6]	0.199	3.34	9.19	0.199	1.67
RIME [6]	0.376	2.14	6.48	0.4	2.35
SBOA [6]	0.199	3.34	9.19	0.199	1.67
CGCRA	0.197954	3.424315	9.031867	0.201218	1.667165

## Data Availability

The data presented in this study are available on request from the corresponding author. The MATLAB R2022b code developed for this study is available from the corresponding author upon reasonable request.
